# Behavioral risk factors and socioeconomic inequalities in ischemic heart disease mortality in the United States: A causal mediation analysis using record linkage data

**DOI:** 10.1371/journal.pmed.1004455

**Published:** 2024-09-17

**Authors:** Yachen Zhu, Laura Llamosas-Falcón, William C. Kerr, Jürgen Rehm, Charlotte Probst

**Affiliations:** 1 Alcohol Research Group, Public Health Institute, Emeryville, California, United States of America; 2 Institute for Mental Health Policy Research, Centre for Addiction and Mental Health, Toronto, Canada; 3 Institute of Medical Science, Faculty of Medicine, University of Toronto, Toronto, Canada; 4 Campbell Family Mental Health Research Institute, Centre for Addiction and Mental Health, Toronto, Canada; 5 Department of Psychiatry, University of Toronto, Toronto, Canada; 6 Dalla Lana School of Public Health, University of Toronto, Toront, Canada; 7 Center for Interdisciplinary Addiction Research (ZIS), Department of Psychiatry and Psychotherapy, University Medical Center Hamburg-Eppendorf (UKE), Hamburg, Germany; 8 PAHO/WHO Collaborating Centre at CAMH, Toronto, Canada & WHO European Region Collaborating Centre at Public Health Institute of Catalonia, Barcelona, Spain

## Abstract

**Background:**

Ischemic heart disease (IHD) is a major cause of death in the United States (US), with marked mortality inequalities. Previous studies have reported inconsistent findings regarding the contributions of behavioral risk factors (BRFs) to socioeconomic inequalities in IHD mortality. To our knowledge, no nationwide study has been conducted on this topic in the US.

**Methods and findings:**

In this cohort study, we obtained data from the 1997 to 2018 National Health Interview Survey with mortality follow-up until December 31, 2019 from the National Death Index. A total of 524,035 people aged 25 years and older were followed up for 10.3 years on average (SD: 6.1 years), during which 13,256 IHD deaths occurred. Counterfactual-based causal mediation analyses with Cox proportional hazards models were performed to quantify the contributions of 4 BRFs (smoking, alcohol use, physical inactivity, and BMI) to socioeconomic inequalities in IHD mortality. Education was used as the primary indicator for socioeconomic status (SES). Analyses were performed stratified by sex and adjusted for marital status, race and ethnicity, and survey year. In both males and females, clear socioeconomic gradients in IHD mortality were observed, with low- and middle-education people bearing statistically significantly higher risks compared to high-education people. We found statistically significant natural direct effects of SES (HR = 1.16, 95% CI: 1.06, 1.27 in males; HR = 1.28, 95% CI: 1.10, 1.49 in females) on IHD mortality and natural indirect effects through the causal pathways of smoking (HR = 1.18, 95% CI: 1.15, 1.20 in males; HR = 1.11, 95% CI: 1.08, 1.13 in females), physical inactivity (HR = 1.16, 95% CI: 1.14, 1.19 in males; HR = 1.18, 95% CI: 1.15, 1.20 in females), alcohol use (HR = 1.07, 95% CI: 1.06, 1.09 in males; HR = 1.09, 95% CI: 1.08, 1.11 in females), and BMI (HR = 1.03, 95% CI: 1.02, 1.04 in males; HR = 1.03, 95% CI: 1.02, 1.04 in females). Smoking, physical inactivity, alcohol use, and BMI mediated 29% (95% CI, 24%, 35%), 27% (95% CI, 22%, 33%), 12% (95% CI, 10%, 16%), and 5% (95% CI, 4%, 7%) of the inequalities in IHD mortality between low- and high-education males, respectively; the corresponding proportions mediated were 16% (95% CI, 11%, 23%), 26% (95% CI, 20%, 34%), 14% (95% CI, 11%, 19%), and 5% (95% CI, 3%, 7%) in females. Proportions mediated were slightly lower with family income used as the secondary indicator for SES. The main limitation of the methodology is that we could not rule out residual exposure-mediator, exposure-outcome, and mediator-outcome confounding.

**Conclusions:**

In this study, BRFs explained more than half of the educational differences in IHD mortality, with some variations by sex. Public health interventions to reduce intermediate risk factors are crucial to reduce the socioeconomic disparities and burden of IHD mortality in the general US population.

## Abstract 2

Yachen Zhu and colleagues leverage data from linked records in the United States and apply causal inference methods to explore how behavioral factors and socioeconomics impact ischaemic heart disease related mortality.

## Author summary

**Why was this study done?:**

- Ischemic heart disease (IHD) is a major cause of death in the United States (US) and worldwide, with high mortality inequalities.
- Conflicting evidence for the contributions of behavioral risk factors (BRFs) to socioeconomic inequalities in IHD mortality exists within international literature, in part because a variety of methodological approaches have been used to answer the question.
- We used a novel counterfactual-based causal mediation method to explore the extent to which smoking, alcohol use, physical inactivity, and BMI explained the socioeconomic inequalities in US IHD mortality.

**What did the researchers do and find?:**

- We obtained data for demographics and health behaviors of 524,025 participants from the 1997 to 2018 US National Health Interview Survey and linked to mortality data from the 2019 National Death Index. We used education as the primary indicator for socioeconomic status (SES).
- We found a clear impact of SES on IHD mortality, with variations by sex. Smoking, physical inactivity, alcohol use, and BMI together explained 74% of the inequalities between low- and high-education groups in males and 61% of the inequalities in females.
- BRFs also explained more than half of the inequalities between middle- and high-education people in both sexes.

**What do these findings mean?:**

- Socioeconomic inequality significantly influences the burden of IHD mortality in the general US population. Public health interventions to target at BRFs are crucial to reducing this disparity.
- The findings are subject to measurement errors as they are based on single, self-reported assessments of the BRFs as well as residual confounding due to unobserved or unknown confounders such as genetic factors that are not accounted for.

## Introduction

Socioeconomic inequalities in ischemic heart disease (IHD) mortality have been observed in different populations around the world over the past few decades [1,2]. Smoking, harmful use of alcohol, leisure-time physical inactivity, and overweight/obesity are important modifiable behavioral risk factors (BRFs) for IHD mortality [3] that tend to cluster among people with low socioeconomic status (SES) [4–6] and have been postulated as the main mechanisms that drive socioeconomic inequalities in IHD mortality [7,8].

Previous studies have investigated the mediating roles of BRFs on educational inequalities in cardiovascular diseases (CVDs) as a whole [9,10], combined IHD morbidity and mortality [11,12], IHD morbidity [13], and IHD mortality [7]. A systematic review reported that a large proportion of the socioeconomic inequalities in cardiovascular health was explained by BRFs, with variations by geographical area, sex, outcome, and SES indicator [8]. The majority of these studies used conventional regression-based methodologies for mediation analyses (i.e., the difference-method [8] and product-of-coefficients method [9]), which do not provide rigorous causal interpretations in survival analyses using proportional hazards models [14] and can lead to severe collider bias when there is uncontrolled mediator-outcome confounder [15]. A few studies employed counterfactual-based methods, which defined indirect (mediation) effect in causal language [15]. For example, Kulhanova and colleagues assessed the impacts of BRFs in educational differences in IHD mortality in 21 European populations by evaluating the population attributable fraction (PAF) under 2 counterfactual scenarios [7]. However, they used a cross-sectional design (collecting risk factors and mortality data at the same period around the year 2000) that could not possibly account for the time-lag between the risk factors and IHD mortality. Using novel counterfactual causal mediation analyses, Petrelli and colleagues investigated the role of BRFs in education inequalities in IHD incidence in Italy, yet without separating IHD mortality from morbidity [12]. The proportion mediated by BRFs ranged from 0% to 87% in the previous literature due to differences in populations, study designs, BRFs included, outcomes, and statistical approaches.

A few United States (US) studies investigating the mediating roles of BRFs in socioeconomic disparities in IHD incidence and mortality had small sample sizes, used the difference-method, and reported inconsistent findings [11,16]. Kittleson and colleagues found that socioeconomic inequalities in IHD incidence and mortality were not explained by BRFs among 1,131 male medical students in The Johns Hopkins Precursors Study [16]. Yet Loucks and colleagues reported that socioeconomic gradients in IHD incidence were largely reduced after adjustment for BRFs among 1,835 participants in the Framingham Heart Study Offspring Cohort [11].

Despite the general success in reducing burden of IHD in the US population, not all people benefited equally in reduction of related BRFs and IHD mortality [17,18]. Notably, no nationwide longitudinal study to date has investigated the extent to which smoking, alcohol use, leisure-time physical inactivity, and overweight/obesity explained socioeconomic inequalities in IHD mortality in the general US population using causal mediation analysis. Particularly, little is known about the contribution of each specific BRF in explaining the growing educational gradients in IHD mortality in the US. Therefore, the aim of this study was to describe the extent to which these 4 BRFs (together and individually) explained socioeconomic inequalities in IHD mortality using a large US national record-linked survey data with the state-of-the-art counterfactual causal mediation method [19,20]. We hypothesized that these BRFs would partially explain the socioeconomic inequalities in IHD mortality.

## Methods

### Ethics statement

All adult participants in the National Health Interview Survey (NHIS) provided written informed consent. Data collection for NHIS and analysis of restricted-use data were approved by the Ethics Review Board (ERB) of the National Center for Health Statistics (NCHS) and the US Office of Management and Budget.

### Data source and measures

We obtained data from the 1997 to 2018 NHIS that were linked to the 2019 National Death Index (NDI) based on both deterministic and probabilistic approaches [21]. Approximately 93% of the total adult sample in NHIS 1997 to 2018, who had sufficient identifying data, were eligible for mortality follow-up [21]. Data linkage was performed by the staff from the NCHS Research Data Center (RDC) following the approval of our RDC research proposal (S1 Analysis Plan). We conducted the data analyses in the RDC in Berkeley, CA. A description of differences between the prospective analysis plan in the protocol and the study performed was documented (S1 Methods). This study followed the Reporting of studies Conducted using Observational Routinely collected health Data (RECORD) Statement (S1 RECORD Statement) and A Guideline for Reporting Mediation Analyses of Randomized Trials and Observational Studies (S1 AGReMA Statement).

We operationalized IHD mortality using the International Classification of Diseases, 10th revision (ICD-10) codes (I20-I25) and 9th revision (ICD-9) codes (410–414). For each participant, the baseline age was calculated as the difference between the interview date and date of birth; the end age was calculated as the difference between date of death and date of birth if he/she was deceased by 12/31/2019, and as the difference between 12/31/2019 and date of birth otherwise. Age at NHIS Interview, age at death or age when last presumed alive, and ICD codes were restricted use variables accessed at the NCHS RDC. We used educational attainment [high school or less (thereafter, low education), some college (middle education), and Bachelor’s degree or more (high education, reference group)] as the primary indicator of SES, and restricted the sample to participants aged 25 years and older assuming that most of the participants had attained their highest level of education by the study enrollment. Education is generally established earlier in the life-course than other social statuses such as occupation, income, and wealth [22], thus it is often regarded as the most important indicator for SES [23]. Besides, education has been regarded as being more stable throughout the life-course and is less likely to be impacted by reverse causation than income [24], that is, income may be more likely to be affected by BRFs such as alcohol use and smoking [25,26]. The categorization of education was informed by Case and Deaton’s works that showed distinct mortality patterns between people with a Bachelor’s degree (high education here) and those without [27–29] as well as previous work on educational inequalities on all-cause mortality [30,31], IHD mortality [32], or adult life expectancy [17] conducted by the team.

Because the educational system in the US had evolved over time in the 20th century, with an increasing trend of college enrollment from the mid-1920s to the end of the century [33], the criteria for high education or high SES defined by education may have changed over time. In sensitivity analysis, we created another set of educational groups using decades-based birth cohort-specific education tertiles. Accounting for survey weights, educational level below or equal to 33% of the decades-based birth cohort was defined as low education, above 33% to below or equal to 67% was defined as middle education, and above 67% was defined as high education. However, education was coded categorically in NHIS with 21 levels from “1: Never attended/kindergarten only” to “21: Doctoral degree,” in which case if a tertile point falls into an educational category with a large sample size, this may lead to an uneven distribution of low, middle, and high education.

Mediators included 4 BRFs. First, alcohol use in the past 12 months was categorized based on the standards of World Health Organization [34] as (1) lifetime abstainer (who never drank alcohol in the past 12 months and never had 12+ drinks in any 1 year, reference group); (2) former drinker (who never drank alcohol in the past 12 months but ever had 12+ drinks in any 1 year); (3) Category I (past year daily average of (0, 20] g for both males and females); (4) Category II (past year daily average of (20,40] g for males and >20 g for females due to smaller number of female drinkers); (5) Category III (past-year daily average of (40, 60] g for males only); and (6) Category IV (past-year daily average >60 g for males only). Second, smoking status was categorized as never smoker (reference group), former smoker (who did not smoke at the time of survey but who ever smoked 100 cigarettes in entire life), current someday smoker, and current everyday smoker in the NHIS questionnaire [35]. Third, body mass index (BMI) was categorized as underweight: <18.5, healthy weight: 18.5 to 24.99 (reference group), overweight: 25 to 29.99, and obesity: ≥30 based on the World Health Organization report on physical status [31,36]. Lastly, leisure-time physical activity was categorized (sedentary: 0 min/week, somewhat active: <150 min/week, and active [reference group]: ≥150 min/week) based on the World Health Organization recommendation of 150 to 300 min of moderate-intensity physical activity per week [31,37].

Potential confounders included marital status (married or living with partner versus never married, widowed, divorced, or separated), race and ethnicity (non-Hispanic black, Hispanic, Others versus non-Hispanic white), and survey year. Age and sex were accounted for in all analyses as outlined below.

### Statistical analysis

Because low SES has been associated with a greater risk of IHD in females than in males [12,38], we first tested for potential interactions between sex and education on IHD mortality using Cox proportional hazards (PH) models with age as the time scale. We performed all following analyses by sex when significant interaction between sex and education was observed. We estimated the associations between education, mediators, and IHD mortality in sex-stratified Cox PH models and tested potential interactions between education and mediators. We checked the PH assumption by evaluating the independence between Schoenfeld residuals and age (the time scale), which did not show any evidence of a violation of the assumption.

**Fig 1:**
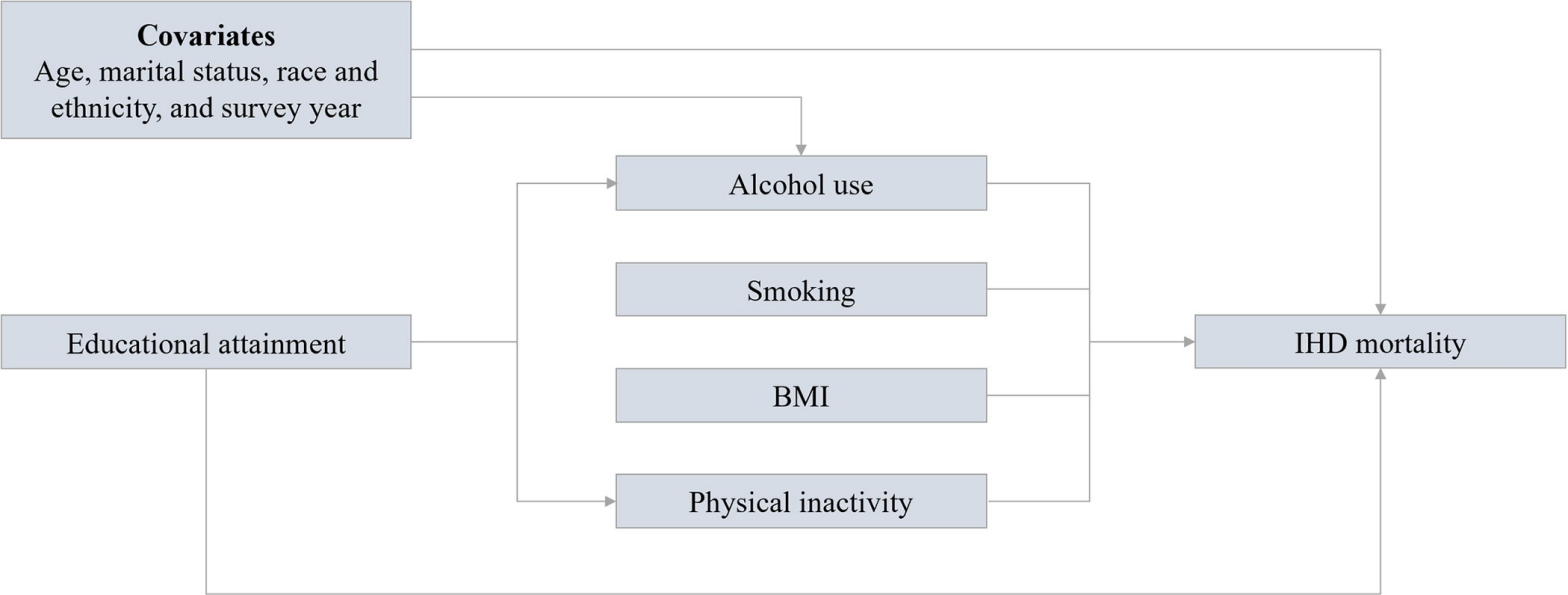
Diagram of the causal pathways between education and IHD mortality. Modified from Puka and colleagues [31]. BMI, body mass index; IHD, ischemic heart disease.

We conducted counterfactual causal mediation analysis [19,20] using inverse probability-weighted marginal structural models to quantify the contributions of all 4 BRFs to educational inequalities in IHD mortality. The method allows for multiple mediators and the decomposition of the total effect of low/middle education (versus high education) into the natural direct effect (NDE) and natural indirect effects (NIEs) mediated through each of the causal pathways (Fig 1) using a flexible underlying statistical model [20]. We used Cox PH model for survival outcomes following the strategy proposed by Lange and colleagues [19,20]. Four auxiliary variables were created to account for the counterfactual levels of the exposure (education) for the 4 indirect causal pathways through mediators, respectively. The original observations in the data set were replicated by *m^K^* times, where *m* = 3 is the number of levels of exposure, and K = 4 is the number of mediators, to account for all combinations of the exposure and auxiliary variables of the exposure [20]. The calculation of mediation weight for observation row *i* in the expanded data is shown below [20,39,40]

$$W_{i}=\prod_{k=1}^{K}\frac{P(M^{k}=M_{i}^{k}|E=E_{i}^{k},C=C_{i})}{P(M^{k}=M_{i}^{k}|E=E_{i}^{0},C=C_{i})},$$

with P indicating the probability of observing a specific level of mediator obtained from multinomial logistic regression of the mediator (M) on the exposure (E) and confounders (C). $M_{i}^{k}$ denotes the value of the *k*th mediator in row *i*. $E_{i}^{k}$ denotes the auxiliary exposure value for mediator *k* in row *i*, whereas $E_{i}^{0}$ denotes the original exposure value in row *i*.

Following the suggestions by NCHS [21], we created the pooled sampling weights for NHIS 1997 to 2018, by computing the mean of the weights for the 22 years of data. The multiplication of the sampling weights and the mediation weights were incorporated into the Cox PH model to derive representative effect estimates of the general US population [39]. We adjusted for marital status, race and ethnicity, and survey year as confounders in the marginal structural causal mediation model described below.

The NDE and NIEs through the mediators represented by hazard ratios (HRs) were parameterized from the marginal structural model—weighted Cox PH model using the expanded data set, in which the hazard function was expressed as follows:

$$\lambda (t|E^{0},M_{E^{1}}^{1},\ldots ,M_{E^{K}}^{K})=\lambda_{0}(t)∙exp(\beta_{0}E^{0}+\sum_{k=1}^{K}\beta_{k}E^{k}+“possible\;interactions”),$$

where *λ*_0_(*t*) is the unspecified baseline hazard describing risk of IHD mortality at reference levels of covariates; *E*^0^ refers to the original exposure values on the direct causal pathway, whereas *E*^1^,…,*E*^*K*^ are auxiliary variables representing the counterfactual values of the exposure on indirect causal pathways through mediators 1 to K; $M_{E^{k}}^{k}$ denotes the *k*th mediator under the counterfactual scenario where the exposure was set to *E*^*k*^ (*k* = 1,…,*K*). The total effect (TE) was calculated as the product of the NDE = exp(*β*_0_) and combined NIE = $\prod_{k=1}^{K}exp(\beta_{k})=exp(\sum_{k=1}^{K}\beta_{k})$ through multiple pathways. Proportions mediated were calculated as $\log\left(NIE)/[\log(NDE)+\log\left(NIE\right)\right]=\sum_{k=1}^{K}\beta_{k}/\left(\beta_{0}+\sum_{k=1}^{K}\beta_{k}\right)$ [20].

Following previous counterfactual-based causal mediation literature [20,41–43], we interpreted the NDE of low education, as the HR comparing the risk of IHD mortality, conditional on the confounders adjusted for, if the education was low with the mediators fixed as if education was high (to what mediator would have been if education had been high), to the risk of IHD mortality if education was high with the mediator fixed at the high education level. We interpreted the NIE of low education as the HR comparing the risk of IHD mortality, conditional on the confounders, if education was low with the mediators fixed as if education was low, to the risk of IHD mortality if education was low with mediators fixed at the high education level.

Assumptions for causal mediation analyses include no uncontrolled confounding, and no mediator-outcome confounder that is itself affected by the exposure [41]. In addition, the multiple-mediator model assumes that different causal pathways must be non-intertwined [20]. We checked this assumption by adding mediators one by one in the mediator models using multinomial logistic regressions and testing the statistical significance of their associations [20,39,40]. When this assumption was violated, we performed a sensitivity analysis including one mediator at a time while adjusting for the 3 others as confounders [31]. In a second sensitivity analysis, we adjusted for exposure–mediator interactions, which allows further decomposition of the indirect effect into differential exposure and differential vulnerability [31,40]. In a third sensitivity analysis, we replaced education with family income as an alternative indicator of SES (classified as high income: ≥400% of poverty threshold [reference group], middle income: 200% to 399% of poverty threshold, low income: <199% of poverty threshold). By using family income as an alternative indicator of SES, we aim to evaluate whether the observed associations persist or vary when a different metric for SES is considered. In a fourth sensitivity analysis, we used SES defined by decades-based birth cohort-specific education tertiles and repeated the main analysis, to evaluate the potential bias incurred by the change of educational systems over time for different birth cohorts.

## Results

During a mean follow-up of 10.3 years, 13,256 deaths by IHD occurred among 524,035 participants (233,543 males and 290,492 females). Descriptive statistics of study participants (unweighted sample sizes and sampling-weighted prevalences) are shown in Table 1. Detailed description of sample characteristics and missing data can be found elsewhere [32]. Given the very few missing data (<5% in each variable), listwise deletion was applied and complete case analysis was performed, assuming that the data are missing at random. The proportion of low education was 41.9% in males and 42.0% in females. The proportion of males with high education was 31.2%, and among females it was 28.9%.

**Table 1:** Characteristics of study participants aged 25 years and older, stratified by sex and education attainment [unweighted sample sizes and weighted mean, standard deviation (SD), and %].

		Males (*N* = 233,543, 48.5%)			Females (*N* = 290,492, 51.5%)		
	Overall	Low education	Middle education	High education	Low education	Middle education	High education
Sample size, *n* (%)^1^	524,035 (100)	101,999 (41.9)	62,465 (26.9)	69,079 (31.2)	129,686 (42.0)	84,018 (29.1)	76,788 (28.9)
Age at survey, mean (SD)	50.3 (16.2)	50.83 (16.3)	48.20 (15.1)	49.03 (15.2)	54.74 (17.5)	49.20 (15.8)	47.15 (14.8)
Years follow up, mean (SD)	10.3 (6.1)	10.26 (6.1)	10.22 (6.1)	10.06 (6.1)	10.53 (6.1)	10.41 (6.1)	9.97 (6.1)
Person-years	5,471,915	1,054,324	642,904	705,130	1,392,978	889,199	787,380
Alcohol use, mean grams per daily (SD) in all participants	5.7 (19.0)	8.97 (29.7)	8.45 (19.5)	7.83 (20.1)	2.38 (12.3)	3.28 (9.7)	4.10 (10.0)
Sample size of current drinkers, n (%)^2^	325,601 (64.7)	62,816 (63.0)	45,503 (73.7)	54,062 (79.2)	54,894 (45.0)	52,742 (64.3)	55,584 (73.4)
Alcohol use, mean grams per day (SD) in current drinkers only	8.95 (23.0)	14.76 (36.6)	12.19 (24.4)	8.95 (23.0)	5.39 (18.9)	5.15 (12.2)	5.64 (11.2)
Alcohol use, *n* (%)^2^							
Lifetime abstainer	160,725 (28.6)	26,915 (25.9)	11,548 (18.4)	11,454 (16.1)	66,192 (48.7)	26,520 (30.3)	18,096 (22.8)
Former drinker	37,709 (6.8)	12,268 (11.1)	5,414 (8.0)	3,563 (4.8)	8,600 (6.3)	4,756 (5.3)	3,108 (3.8)
Category I: (0, 20] g/day	287,928 (57.3)	50,123 (50.7)	37,607 (61.6)	46,321 (68.4)	51,582 (42.3)	49,792 (60.8)	52,503 (69.4)
Category II: (20, 40] g/day for males; >20 g/day for females	26,742 (5.3)	6,859 (6.7)	4,848 (7.5)	5,692 (8.0)	3,312 (2.7)	2,950 (3.5)	3,081 (4.0)
Category III: (40, 60] g/day for males only	5,687 (1.1)	2,722 (2.6)	1,602 (2.5)	1,363 (1.9)	0 (0.0)	0 (0.0)	0 (0.0)
Category IV: >60 g/day for males only	5,244 (1.0)	3,112 (3.0)	1,446 (2.1)	686 (0.9)	0 (0.0)	0 (0.0)	0 (0.0)
Smoking, *n* (%)^2^							
Never smoker	292,254 (56.0)	40,283 (39.9)	28508 (47.0)	43,860 (65.0)	76,565 (57.8)	47,980 (57.6)	55,058 (72.9)
Former smoker	127,703 (24.6)	31,454 (30.2)	18,966 (30.2)	18,003 (25.7)	24,929 (19.8)	19,105 (22.8)	15,246 (19.3)
Current someday smoker	21,401 (3.8)	5,466 (5.1)	3227 (4.8)	2,414 (3.2)	4,566 (3.3)	3,565 (3.9)	2,163 (2.6)
Current everyday smoker	82,677 (15.6)	24,796 (24.8)	11,764 (17.9)	4,802 (6.2)	23,626 (19.2)	13,368 (15.7)	4,321 (5.2)
BMI, *n* (%)^2^							
Underweight	8,724 (1.5)	986 (0.9)	348 (0.5)	338 (0.4)	3,105 (2.3)	1,798 (2.1)	2,149 (2.7)
Healthy weight	181,061 (34.1)	27,778 (26.1)	15,745 (23.9)	22,368 (31.1)	44,566 (34.8)	31,488 (38.1)	39,116 (52.0)
Overweight	187,626 (36.3)	43,255 (42.1)	27,148 (43.7)	31,728 (46.4)	40,868 (31.1)	24,565 (29.1)	20,062 (26.2)
Obese	146,624 (28.1)	29,980 (30.9)	19,224 (31.9)	14,645 (22.0)	41,147 (31.7)	26,167 (30.7)	15,461 (19.1)
Physical inactivity, *n* (%)^2^							
Active	226,244 (45.0)	35,614 (35.9)	31,910 (51.5)	44,119 (64.0)	35,210 (28.8)	35,561 (43.5)	43,830 (57.6)
Somewhat active	94,868 (18.5)	15,157 (15.2)	10,459 (17.2)	11,521 (17.1)	23,540 (18.8)	18,174 (22.1)	16,017 (21.3)
Sedentary	202,923 (36.5)	51,228 (48.9)	20,096 (31.4)	13,439 (18.8)	70,936 (52.4)	30,283 (34.4)	16,941 (21.1)
Race/ethnicity, n (%)^2^							
White	342,084 (70.7)	59,656 (64.0)	44,173 (73.8)	52,566 (78.5)	72,952 (64.1)	56,500 (72.8)	56,237 (76.3)
Black	72,601 (11.3)	15,061 (12.4)	8,232 (11.5)	5,266 (6.7)	21,970 (13.5)	14,028 (13.5)	8,044 (8.5)
Hispanic	81,415 (12.6)	23,706 (20.1)	7,308 (10.5)	4,685 (6.0)	29,695 (18.0)	10,208 (9.7)	5,813 (6.2)
Other	27,935 (5.5)	3,576 (3.5)	2,752 (4.2)	6,562 (8.9)	5,069 (4.3)	3,282 (3.9)	6,694 (9.0)
Income, *n* (%)^2^							
Low	142,955 (22.4)	36,720 (32.1)	11,650 (16.1)	5,926 (6.9)	58,024 (37.8)	22,929 (22.4)	7,706 (8.0)
Middle	128,998 (25.0)	28,487 (28.9)	18,603 (29.5)	12,596 (17.3)	28,502 (25.0)	24,335 (29.2)	16,475 (19.5)
High	157,087 (34.7)	17,862 (20.2)	22,091 (38.3)	39,382 (59.8)	15,710 (16.1)	22,013 (31.1)	40,029 (56.3)
Missing	94,995 (17.9)	18,930 (18.8)	10,121 (16.1)	11,175 (16.0)	27,450 (21.1)	14,741 (17.4)	12,578 (16.2)
Marital status, *n* (%)^2^							
Married/cohabitating	286,008 (67.9)	60,284 (70.1)	36,578 (70.8)	44,340 (76.5)	59,474 (59.2)	41,407 (63.2)	43,925 (70.7)
Not married/cohabitating	238,027 (32.1)	41,715 (29.9)	25,887 (29.2)	24,739 (23.5)	70,212 (40.8)	42,611 (36.8)	32,863 (29.3)

^1^ Percentages of the overall sample and educational levels among male and female participants, respectively.

^2^ Percentages by column.

The sampling-weighted prevalence of never smokers was highest among males with high education (65.0%; unweighted *n* = 43,860 never smokers among 69,079 males with high education), and lowest among males with low education (39.9%; unweighted *n* = 40,283 never smokers among 101,999 males with low education); the sampling-weighted prevalence of current everyday smokers was highest among males with low education (24.8%; unweighted *n* = 24,796 out of 101,999 males with low education), and lowest among males with high education (6.2%; *n* = 4,802 among 69,079 males with high education). Similarly, the prevalence of never smokers was highest among females with high education (72.9%; *n* = 55,058 among 76,788 females with high education), and was similar in those with low (57.8%; *n* = 76,565 among 129,686 females with low education) and middle education (57.6%; *n* = 47,980 among 84,018 females with middle education); the prevalence of current everyday smokers was highest among females with low education (19.2%; *n* = 23,626 among 129,686 females with low education), and lowest among females with high education (5.2%; *n* = 4,321 among 76,788 females with high education).

In both males and females, the prevalence of being physically active was highest among participants with high education (64.0% and 57.6%, respectively), and lowest among participants with low education (35.9% and 28.8%); the prevalence of being sedentary was highest among participants with low education (48.9% and 52.4%), and lowest among participants with high education (18.8% and 21.1%). Similar distributions were observed for BMI.

Regarding alcohol use, different from the distributions of the other BRFs, the prevalence of lifetime abstainers was highest among low-education males (25.9%) and females (48.7%). The prevalence of Category I and II drinkers was highest among high-education males (68.4% and 8.0%, respectively) and females (69.4% and 4.0%). However, the prevalence of Category III and IV were slightly higher in low-education males (2.6% and 3.0%) than in middle (2.5% and 2.1%) and high-education males (1.9% and 0.9%). S1 Fig shows the sampling-weighted prevalence of the highest BRFs categories by sex and education.

We observed a significant interaction between sex and education, showing that the educational inequality (low versus high education) in IHD mortality in females was significantly higher than males (*p* < 0.001) (S1 Table). We therefore conducted all statistical analyses by sex. Proportional hazards assumptions were met based on statistical tests (S2 Table). In both males and females, compared to those with high education, the risk of IHD mortality was statistically significantly higher in people with low and middle education (Table 2). There was a larger excess risk associated with low education (comparing with high education) in females (HR = 2.05, 95% CI: 1.84, 2.30) than in males (HR = 1.86, 95% CI: 1.72, 2.00). The associations were attenuated after adjustment for alcohol use, smoking, BMI, and physical inactivity (Table 2). Smoking (current or former versus never), underweight and obese (versus healthy weight), and being sedentary or only somewhat active (versus active) are all significant predictors of higher IHD mortality (Table 2). We examined the interactions between education and each of the 4 risk factors. There was only a significant interaction between alcohol use and education in both sexes, showing that the protective association of drinking ≤20 grams per day with IHD mortality was stronger in people with high education than those with low education (S3 Table). However, there were no consistent interactions of education with smoking (S4 Table) or physical inactivity (S5 Table), and there was no interaction between education and BMI in either sex (S6 Table).

**Table 2:** Associations between education, alcohol use, smoking, BMI, physical inactivity, and ischemic heart disease mortality in sex-stratified Cox proportional hazards models.

	Males						Females					
	Minimally adjusted^1^			Fully adjusted^2^			Minimally adjusted^1^			Fully adjusted^2^		
	HR^3^	95% CI^4^	*p*-Value^5^	HR^3^	95% CI^4^	*p*-Value^5^	HR^3^	95% CI^4^	*p*-Value^5^	HR^3^	95% CI^4^	*p*-Value^5^
Education												
Low	1.86	(1.72, 2)	<0.001	1.32	(1.22, 1.42)	<0.001	2.05	(1.84, 2.3)	<0.001	1.52	(1.36, 1.71)	<0.001
Middle	1.64	(1.5, 1.79)	<0.001	1.33	(1.22, 1.45)	<0.001	1.61	(1.42, 1.83)	<0.001	1.38	(1.22, 1.57)	<0.001
High	ref			ref			ref			ref		
Marital status												
Not married/cohabitating	ref			ref			ref			ref		
Married/cohabitating	0.63	(0.6, 0.67)	<0.001	0.67	(0.64, 0.71)	<0.001	0.64	(0.59, 0.69)	<0.001	0.71	(0.66, 0.76)	<0.001
Race and ethnicity												
White	ref			ref			ref			ref		
Black	0.94	(0.85, 1.03)	0.167	0.87	(0.79, 0.96)	0.005	1.15	(1.04, 1.27)	0.005	1.02	(0.92, 1.13)	0.708
Hispanic	0.74	(0.67, 0.82)	<0.001	0.76	(0.69, 0.84)	<0.001	0.79	(0.7, 0.89)	<0.001	0.77	(0.69, 0.87)	<0.001
Other	0.74	(0.61, 0.9)	0.002	0.73	(0.6, 0.89)	0.002	0.71	(0.59, 0.85)	<0.001	0.74	(0.61, 0.89)	0.002
Alcohol use												
Lifetime abstainer				ref						ref		
Former drinker				1.04	(0.95, 1.13)	0.368				1.07	(0.96, 1.19)	0.223
Category I: (0, 20] g/day				0.74	(0.69, 0.8)	<0.001				0.66	(0.61, 0.71)	<0.001
Category II: (20, 40] g/day for males; >20 g/day for females				0.74	(0.65, 0.84)	<0.001				0.62	(0.49, 0.77)	<0.001
Category III: (40, 60] g/day for males only				0.91	(0.76, 1.09)	0.316				-		
Category IV: >60 g/day for males only				1.08	(0.87, 1.32)	0.494				-		
Smoking												
Never smoker				ref						ref		
Former smoker				1.41	(1.31, 1.52)	< .001				1.42	(1.33, 1.53)	<0.001
Current someday smoker				1.75	(1.48, 2.07)	< .001				1.99	(1.62, 2.44)	<0.001
Current everyday smoker				2.43	(2.23, 2.65)	< .001				2.33	(2.11, 2.57)	<0.001
BMI												
Underweight				1.51	(1.16, 1.96)	0.002				1.29	(1.07, 1.56)	0.009
Healthy weight				ref						ref		
Overweight				0.97	(0.9, 1.04)	0.423				1.02	(0.95, 1.1)	0.543
Obese				1.37	(1.26, 1.48)	<0.001				1.29	(1.2, 1.39)	<0.001
Physical inactivity												
Active				ref						ref		
Somewhat active				1.28	(1.17, 1.4)	<0.001				1.32	(1.18, 1.47)	<0.001
Sedentary				1.61	(1.5, 1.72)	<0.001				1.83	(1.67, 2)	<0.001

Note: All models adjusted for categorical survey year.

^1^ Minimally adjusted model adjusted for marital status, race and ethnicity, and survey year.

^2^ Fully adjusted model adjusted for marital status, race and ethnicity, survey year, alcohol use, smoking, BMI, and physical inactivity.

^3^ HR: hazard ratio.

^4^ CI: confidence interval.

^5^ *p*-Values were obtained from Wald tests.

We found statistically significant NDE of SES and NIE mediated through the pathways of BRFs conditional on confounders, as shown in Table 3. In males, the NIE of low education (versus high education) was HR = 1.51, 95% CI: 1.45, 1.57 (74%, 95% CI: 63%, 88%, of the total effect), among which 29% (95% CI: 24%, 35%) was mediated through smoking (HR = 1.18, 95% CI: 1.15, 1.20), 27% (95% CI: 22%, 33%) through physical inactivity (HR = 1.16, 95% CI: 1.14, 1.19), 12% (95% CI: 10%, 16%) through alcohol use (HR = 1.07, 95% CI: 1.06, 1.09), and 5% (95% CI: 4%, 7%) through BMI (HR = 1.03, 95% CI: 1.02, 1.04); the NIE of middle education (versus high education) was HR = 1.27, 95% CI: 1.24, 1.30 (56%, 95% CI: 45%, 74%, of the total effect), 25% (95% CI: 19%, 33%) was mediated through smoking (HR = 1.11, 95% CI: 1.10, 1.13), 18% (95% CI: 14%, 24%) through physical inactivity (HR = 1.08, 95% CI: 1.07, 1.09), 8% (95% CI: 6%, 12%) via alcohol use (HR = 1.04, 95% CI: 1.03, 1.04), and 6% (95% CI: 4%, 8%) via BMI (HR = 1.02, 95% CI: 1.02, 1.03). In females, the NIE of low education was HR = 1.47, 95% CI: 1.40, 1.54 (61%, 95% CI: 49%, 79%, of the total effect), among which 26% (95% CI: 20%, 34%) was mediated through physical inactivity (HR = 1.18, 95% CI: 1.15, 1.20), 16% (95% CI: 11%, 23%) via smoking (HR = 1.11, 95% CI: 1.08, 1.13), 14% (95% CI: 11%, 19%) via alcohol use (HR = 1.09, 95% CI: 1.08, 1.11), and 5% (95% CI: 3%, 7%) BMI (HR = 1.03, 95% CI: 1.02, 1.04); the NIE of middle education was HR = 1.25 (57%, 95% CI: 40%, 93%, of the total effect), among which 22% (95% CI: 15%, 36%) was mediated through physical inactivity (HR = 1.09, 95% CI: 1.08, 1.10), 19% (12, 34%) smoking (HR = 1.08, 95% CI: 1.06, 1.09), 10% (95% CI: 6%, 17%) via alcohol use (HR = 1.04, 95% CI: 1.03, 1.04), and 6% (95% CI: 3, 12%) via BMI (HR = 1.03, 95% CI: 1.02, 1.03).

**Table 3:** Natural direct and indirect effects (hazard ratio scale) of education on ischemic heart disease mortality operating via the pathways of alcohol use, smoking, BMI, and physical inactivity.

	Males		Females	
	HR^1^ (95% CI^2^)	% TE (95% CI)^3^	HR^1^ (95% CI)	% TE (95% CI)^3^
*Low education vs*. *high education*				
Natural direct effect (NDE)	1.16 (1.06, 1.27)	26 (12, 38)	1.28 (1.1, 1.49)	39 (19, 53)
Natural indirect effect (NIE)	1.51 (1.45, 1.57)	74 (63, 88)	1.47 (1.4, 1.54)	61 (49, 79)
Alcohol use	1.07 (1.06, 1.09)	12 (10, 16)	1.09 (1.08, 1.11)	14 (11, 19)
Smoking	1.18 (1.15, 1.2)	29 (24, 35)	1.11 (1.08, 1.13)	16 (11, 23)
BMI	1.03 (1.02, 1.04)	5 (4, 7)	1.03 (1.02, 1.04)	5 (3, 7)
Physical inactivity	1.16 (1.14, 1.19)	27 (22, 33)	1.18 (1.15, 1.2)	26 (20, 34)
Total effect (TE)	1.76 (1.62, 1.9)		1.88 (1.66, 2.13)	
*Middle education vs*. *high education*				
Natural direct effect (NDE)	1.2 (1.09, 1.33)	44 (25, 56)	1.18 (1.01, 1.39)	43 (3, 61)
Natural indirect effect (NIE)	1.27 (1.24, 1.3)	56 (45, 74)	1.25 (1.21, 1.28)	57 (40, 93)
Alcohol use	1.04 (1.03, 1.04)	8 (6, 12)	1.04 (1.03, 1.04)	10 (6, 17)
Smoking	1.11 (1.1, 1.13)	25 (19, 33)	1.08 (1.06, 1.09)	19 (12, 34)
BMI	1.02 (1.02, 1.03)	6 (4, 8)	1.03 (1.02, 1.03)	6 (3, 12)
Physical inactivity	1.08 (1.07, 1.09)	18 (14, 24)	1.09 (1.08, 1.1)	22 (15, 36)
Total effect (TE)	1.53 (1.39, 1.69)		1.47 (1.27, 1.71)	

^1^ HR: hazard ratio.

^2^ CI: confidence interval.

^3^ Proportions mediated were calculated as log(NIE)/[log(NDE)+log(NIE)].

Because we found significant associations between mediators in multinomial logistic regressions, in sensitivity analysis, we evaluated one mediator at a time in causal mediation analyses and adjusting for the other 3 as confounders. We observed slightly higher proportion mediated through each of the causal pathways (S7 Table) than when including all 4 BRFs as mediators in the model (Table 3).

Because of the significant interaction between alcohol use and education in both sexes (S3 Table), we conducted another sensitivity analysis further decomposing the indirect effects into differential exposure and differential vulnerability on the pathway of alcohol use. We found that the indirect effects of low and middle education on IHD mortality via alcohol use could be largely attributed to differential exposure of alcohol use in people with different educational attainments, yet without statistically significant differential vulnerabilities (S8 Table).

Using family income as SES, the proportions mediated were lower (S9 Table) than those using education as SES (Table 3), likely because there was a large missing category in income, shrinking the sample sizes of the high, middle, and low categories of income compared with those of education.

Using educational groups defined by birth cohort-specific education tertiles led to more evenly distributed average baseline age by educational groups (S10 Table); it also led to a more even distribution of educational groups for birth cohorts born before 1940 with a less even distribution of educational levels for birth cohorts born after 1970 due to tertile points falling into large educational categories (S11 Table). Proportional hazards assumptions were still supported (S12 Table). The educational inequalities estimated from the Cox PH models (S13 Table) were slightly attenuated, compared to the original results (Table 2), and yet remained strong and statistically significant. Despite these changes, we derived largely robust results from the updated causal mediation analysis (S14 Table), similar to those from the main analysis (Table 3) that used the uniformly defined educational groups, demonstrating that changes of the education system in the US in the past century had limited impacts on our findings regarding the extent to which socioeconomic inequalities were explained by BRFs.

## Discussion

Our analyses disentangled the intricate relationship between SES, BRFs, and IHD mortality in a large US national adult sample. Socioeconomic inequalities in IHD mortality were identified with both SES indicators: individual education and family income. The causal mediation decomposition provided insight into both the direct and indirect pathways through which low and middle SES contributed to IHD mortality compared with high SES, offering valuable information to understand the mechanisms behind the socioeconomic inequalities in IHD mortality in the US during the past 2 decades. Overall, BRFs contributed to more than half of the educational disparities in IHD mortality for both males and females, suggesting that socioeconomic disparities in the risk of IHD mortality can be mostly attributed to differential distributions of smoking and leisure-time physical inactivity, followed by alcohol use and BMI, across SES subgroups. The largest proportion mediated (74%) through BRFs was identified between low (versus high) education groups and IHD mortality among males, with 29% attributed to smoking, 27% to physical inactivity, 12% to alcohol, and 5% to BMI. In females, the corresponding mediation proportion was 61%, with 26% attributed to physical inactivity, followed by 16% attributed to smoking, 14% to alcohol use, and 5% to BMI. Besides, BRFs explained up to 42% and 46% of the income disparities in IHD mortality in males and females, respectively.

Comparisons of our study with previous studies are not straightforward given the different endpoints (IHD/CVD, morbidity/mortality), sets of BRFs, SES indicators, study designs, methodologies, and populations investigated. However, consistent with a systematic review on IHD morbidity [38], our study found a larger educational inequality in IHD mortality for females than for males. The sex differences observed in our study are well supported by 2 previous longitudinal studies in the US: the first one using the 1971 to 1993 National Health and Nutrition Examination Survey found that less than high school education (compared to college education) was associated with a stronger risk of IHD in females (hazard ratio = 2.15, 95% CI: 1.46 to 3.17) than in males (hazard ratio = 1.58, 95% CI: 1.18 to 2.12) [44]; another study using the 2006 Health and Retirement Study for adults aged 51 years and older in the US also suggested a more pronounced association between SES and cardiovascular risk in females than in males [45]. Additionally, our sex-specific results are similar to an Italian longitudinal study [12], in which the educational inequalities in CVD and IHD incidence were stronger among females than in males and BRFs explained a larger proportion of such educational inequalities in males, with smaller mediating effects found in females.

In our study, for female participants the total proportion of educational inequalities in IHD mortality mediated by smoking, alcohol use, physical inactivity, and BMI (61%) was lower than the proportion of 87% found in a prospective study of 1.2 million UK females [46]—likely due to the differences in populations and methodologies for mediation analysis. Our proportion mediated in male participants (74%) was also lower than the proportion of 84% in male found in an Italian National Health Interview Survey study, in which a slightly different set of mediators were considered that replaced alcohol use with diabetes and hypertension [12]. However, our proportion mediated was larger than the 36% estimated from a mendelian randomization analysis using UK Biobank data, in which smoking, BMI, and systolic blood pressure were mediators [9], and much higher than the proportion of 15% reported by a Sweden study [10], in which CVD mortality was the outcome, father’s occupational social class (manual versus non-manual) was the SES indicator, mediators included diet in addition to those examined in our study. Overall, comparisons with previous studies are complicated due to different study designs, populations, methods, as well as different SES indicators, mediators, and endpoints.

Despite the above complexities, consistent with the systematic review by Petrovic and colleagues on all-cause mortality and cardiovascular diseases [8], we found that smoking contributed to a large proportion of socioeconomic inequalities in IHD mortality in the US, explaining 29% of the differences in IHD mortality risk between low- and high-education people in males and 16% in females. Behind these numbers, smoking acting as an important causal pathway between SES and IHD mortality is well supported by the higher prevalence of cigarette use among socioeconomically disadvantaged groups, especially in male [5]. Smoking serves as a more socially acceptable means to cope with stress, regulate mood, and address daily challenges linked to adverse social conditions in people with low SES than those with high SES [47]. Biologically, it has been well founded that chemicals in cigarettes can cause the cells in blood vessels to become swollen and inflamed, which would narrow the blood vessels and form clots inside veins and arteries, leading to higher rates of IHD [48]. The harmful effects of smoking could be further exacerbated when combined with the higher rates of leisure-time physical inactivity and obesity in socioeconomically disadvantaged people [49,50].

In our study, leisure-time physical inactivity explained 27% of the socioeconomic inequality (between low and high education) in IHD mortality in male and 26% in females. Since habits of physical activity tend to develop from childhood, adolescence, to adulthood and can track over the life-course [51,52], promotion of physical activity through early education in schools may lay the foundation for activity habits in later life that contribute to better cardiovascular health [53]. Different from leisure-time physical activity investigated in our study, studies have found that occupational physical activity did not provide beneficial association with cardiovascular mortality [54], despite the relatively higher prevalence of occupational physical activity in people with low SES [55]. This highlighted the importance of promoting leisure-time physical activity particularly in people with low SES from early education to reduce burdens of IHD mortality [56].

Alcohol and BMI were found to mediate smaller yet still significant proportions of the total effect of SES in both sexes. The indirect effects of SES through alcohol use could be attributed to differences in drinking volumes as well as patterns across heterogeneous SES groups. Studies have showed harmful associations of highly frequent drinking with cardiovascular mortality only in the low SES people [57] yet poorer protective associations of low-to-moderate drinking with IHD morality in the low compared to the high SES group [32]. These socioeconomic differences highlight the need for implementing effective alcohol policies aimed at increasing prices and reducing physical availability [58]. BMI and obesity are closely related to physical inactivity and would benefit from policies and interventions addressing sedentary lifestyles. Maintaining healthy diets [59] as well as equal access to newer treatments for obesity [60] are also major contributors to normal BMI and there is a need for policies and interventions aimed at equity across all socioeconomic groups.

Similar to Mehta and colleagues, we found that BRFs explained a larger proportion of educational inequalities than income inequalities [61]. Apart from the different categorizations and a missing group in income, this difference could also be attributed to the stronger social patterning of BRFs across educational groups than income groups [62]. Our findings that smoking contributed the most to educational disparities in IHD mortality in male while physical inactivity contributed the most to the disparities in females are supported by the fact that smoking is more prevalent in males [63] and that less females met the physical activity guidelines than males in the US [64], suggesting sex-tailored prevention and treatment efforts for low-education people. When using family income as the SES, we found that physical inactivity consistently explained the largest proportion of income inequalities in IHD mortality in both sexes. This may be explained by the fact that physical activity habits tend to be developed from childhood, adolescence, to adulthood [51] and can be impacted by living conditions from childhood that is closely linked to family income in both sexes [65].

This study has several major strengths. First, we applied a counterfactual causal mediation methodology with inverse probability-weighted marginal structural models [19,20], which provides rigorous causal interpretations in survival analyses [14] and can account for potential exposure–mediator interactions [31,40]. Although some previous studies have investigated the role of BRFs in the relationship between SES and IHD mortality, the majority of them applied the conventional regression-based difference-method or product-method [8], which do not have any sort of clear causal interpretation as a measure of effect in the general setting of non-rare outcome with proportional hazards models [14] and can lead to severe collider bias when there is a prominent mediator-outcome confounder left uncontrolled [15]. Instead, reliable identification of causal mechanism in mediation analysis requires the concept of natural direct and indirect effects with a counterfactual strategy [15], which is what we applied in this study. Besides, the method can incorporate multiple mediators in survival analysis and is mathematically consistent [19,20]. Second, prior to our analyses, very few studies have been performed in the US [11,16] with no nationwide study conducted in the general US population, which remained an important knowledge gap. In our analyses using a large mortality linked NHIS data with 524,025 participants, we accounted for survey weights, making our results representative of the general US population. Third, our longitudinal study design with an average follow-up of 10.3 years can better inform the causal relationship between SES, BRFs, and IHD mortality than cross-sectional studies. Fourth, we used multiple indicators for SES, both education and family income: Education affects cognitive abilities and trains people to obtain, evaluate, and utilize information that leads to desirable health outcomes [22], whereas family income impacts health through social and geographic environments, living conditions, and social norms on health practices in the surroundings. Using both indicators in our analyses provided a more comprehensive picture of different dimensions of socioeconomic gradient in IHD mortality and how they were explained by BRFs. Additionally, to account for the impact of evolution of educational system in the US in the 20th century, with an increasing trend of college enrollment from the mid-1920s to the end of the century [33], we created another set of educational groups based on decades-based birth cohort-specific education tertiles, which showed largely robust results.

Despite the strengths noted above, our results should be cautiously interpreted with several limitations. First, we did not evaluate the mediating effects of health care access or insurance status [66]. A systematic review found strong socioeconomic inequalities in access to treatment of IHD, especially coronary angiography; differential access to drug treatment and cardiac rehabilitation by SES were also found in half of the included studies. Notably, such disparities were stronger in countries without universal health coverage (UHC) such as the US, to the disadvantage of individuals with low SES [67]; while access to treatment was less often to differ by SES in countries with UHC, where mediators such as BRFs likely accounted for most of the socioeconomic gradient in CVD [68]. The enforcement of Affordable Care Act (ACA) in 2014 aimed at moving the US closer to UHC by expanding health coverage for millions of Americans across SES, age, race, and ethnicity, via Medicaid expansion, launch of health insurance marketplace for private coverage, etc. [69]. Studies have demonstrated a significant reduction of socioeconomic disparities in CVD preventive care utilization from 2011 to 2017 following the ACA [70]. However, our analyses covered a longer period from 1997 to 2019, and health care access information was only collected at the survey interview in NHIS, which could not possibly account for the increased utilization of CVD-related preventive services after ACA for participants enrolled in NHIS cycles before 2014. Furthermore, health care access and insurance-related variables were not included in our data application (S1 Analysis Plan) and restricted use data at the RDC. Future US studies should evaluate how access to health care contributed to the socioeconomic inequalities in IHD mortality beyond the proportions explained by BRFs before and after ACA. Other studies considered blood pressure, health status/conditions, and diet as mediators [8,9,71]; however, these variables are closely linked to the existing BRFs evaluated in our analyses [7]. For example, the effect of diet is likely to act via BMI, thus including BMI in the model is likely to have already captured some of the mediation effect through diet. To keep our models parsimonious, reasonably within the computational limitation [20], and adhere to the assumption of non-intertwined mediators as much as possible [20], we did not further include blood pressure, diet, health status or conditions as mediators. Second, BRFs were self-reported. It is important to note that alcohol use and smoking tend to be underreported in general, although the validity of self-reporting has been found similar across socioeconomic groups in the literature [72–74]. Previous research has found that non-differential measurement error of mediators can lead to an underestimation of the indirect effects using the traditional difference-method [75]. However, in our setting, counterfactual levels of SES were entered into the marginal structural modeling as proxies of mediators to approximate what would be observed under the counterfactual level of SES, and the mediation weights accounted for the likelihood of mediator levels that would be observed under the counterfactual SES [20], which is unlikely to bias the indirect effect as much as the traditional methods where causal interpretations are less clear [10,76]. Besides, we did not account for the time-varying effects of BRFs [77], which may have led to an underestimation of the mediated effects [78]. Carter and colleagues found larger mediation effects estimated from mendelian randomization than the observational method [9], indicating that using genetic instruments to proxy the exposure (education) and mediators may be more robust to non-differential measurement error and confounding than using one snapshot of risk factors in observational studies [79]. However, Franks and colleagues found that accounting for the temporal changes explained little of the socioeconomic inequalities in IHD in an observation study [80]. Future US studies should triangulate evidence from different methods to better infer causal relationships. Third, we assumed that the covariates included in the models (age, race and ethnicity, marital status, and survey year) were sufficient to control for the exposure-outcome, mediator-outcome, and exposure-mediator confounding, and that none of the mediator-outcome confounders are themselves affected by the exposure [41], yet in our observational study we cannot rule out the possibility of residual confounding and ensure that BRFs-IHD mortality confounders (for example, marital status) are not affected by SES. Unobserved/unknown confounders related to both the mediators and IHD mortality, such as genetic factors related to both obesity and IHD, may have biased our results. However, a recent study found that the risk of developing CVD is lower in people with obesity who have a genetic predisposition for high BMI than those whose obesity was mainly influenced by environmental factors such as lifestyle [81]. With other lifestyle/behavioral factors (alcohol use, smoking, physical activity) included in our analyses as other mediators, and race and ethnicity and marital status adjusted for as key confounders based on previous literature [7–9,11,12,16,38,44,46], we expect that the number of such unobserved/unknown confounding factors will likely be small and less prominent than the existing variables included in our models. Besides, although our categorization of education was informed by Case and Deaton’s landmark works on educational differences in mortality in the US [23,27–29] and previous literature on socioeconomic gradient in mortality [30,31], and our categorizations of mediators were informed by World Health Organization’s standards [34,36,37], which led to meaningful and easily interpretable results, information may be lost in such categorizations, and proportions mediated may be underestimated. Lastly, 2 dimensions of alcohol use have been shown to impact on IHD incidence and mortality, both average level of consumption and frequency of heavy drinking occasions [82]. However, in the current analyses, we had to restrict ourselves to 1 dimension—average level of alcohol consumption, which may in part explain the relatively lower contribution of alcohol use, especially among male participants.

In summary, our results highlight the need for attention to implementing effective policies and interventions addressing each of these behaviors both separately and together. As unhealthy behaviors usually cluster among individuals with low SES [4–6], while individuals with high SES tend to exhibit higher awareness and place greater importance on preventive health measures, in addition to demonstrate a greater capacity to sustain healthy behaviors over time, public health policies should account for socioeconomic backgrounds when designing and implementing cost-effective interventions. The “best buys” of the World Health Organization for reducing noncommunicable disease [83] should be considered with priority, as these interventions are specific for BRFs, have shown to be cost-effective and easy to implement in reducing mortality inequalities [84,85]. Stakeholders and clinicians should incorporate behavioral factors into risk assessments and screening tools, involving the mitigation of BRFs through effective and equitable primary and secondary prevention measures, as well as providing health education and training on condition management and increasing awareness on health behaviors. Furthermore, our results also highlight the sex differences in BRFs and SES gradients, suggesting important implications for the development and targeting of preventive measures for IHD. Preventive strategies to reduce the prevalence of smoking and promote leisure-time physical activity among low-SES males and females, respectively, are likely to substantially reduce the socioeconomic disparities in IHD mortality in the US. Public health campaigns aimed at raising awareness about cardiovascular health could customize messaging and outreach efforts to effectively reach males and females, considering differences in risk perceptions and health-seeking behaviors.

## Supporting information

S1 Analysis Plan Evaluation of the extent to which the association between socioeconomic status and ischemic heart disease mortality is mediated by health behaviors. (DOCX)

S1 Methods Description of differences between the analysis plan and the study performed. (DOCX)

S1 RECORD Statement The Reporting of studies Conducting using Observational Routinely collected health Data (RECORD) Statement. (DOCX)

S1 AGReMA Statement A Guideline for Reporting Mediation Analyses. (DOCX)

S1 Fig Sampling-weighted prevalence of behavioral risk factors by sex and education. (DOCX)

S1 Table Hazard ratios from Cox proportional hazards model with interaction between education and sex. (DOCX)

S2 Table Tests of proportional hazards assumptions for Cox proportional hazards models in Table 2. (DOCX)

S3 Table Interaction effects between education and alcohol use on ischemic heart disease mortality by sex. (DOCX)

S4 Table Interaction effects between education and smoking on ischemic heart disease mortality by sex. (DOCX)

S5 Table Interaction effects between education and physical inactivity on ischemic heart disease mortality by sex. (DOCX)

S6 Table Interaction effects between education and BMI on ischemic heart disease mortality by sex. (DOCX)

S7 Table Sensitivity Analysis 1: Causal mediation analyses evaluating one mediator at a time. (DOCX)

S8 Table Sensitivity Analysis 2: Natural direct and indirect effects (hazard ratio scale) of education on ischemic heart disease mortality operating via the pathways of alcohol use, smoking, BMI, and physical inactivity, with decomposition of indirect effects into differential exposure and differential vulnerability on the pathway of alcohol use. (DOCX)

S9 Table Sensitivity Analysis 3: Natural direct and indirect effects (hazard ratio scale) of family income on ischemic heart disease mortality operating via the pathways of alcohol use, smoking, BMI, and physical inactivity. (DOCX)

S10 Table Descriptive statistics of study participants aged 25 years and older, stratified by sex and decades-based birth cohort-specific education tertiles (unweighted sample sizes and weighted mean, SD, and %). (DOCX)

S11 Table Sample size (unweighted *n*) and proportion (weighted %) by decades-based birth cohort and educational level. (DOCX)

S12 Table Tests of proportional hazards assumptions for Cox proportional hazards models using education defined by decades-based birth cohort-specific education tertiles. (DOCX)

S13 Table Associations between education defined by decades-based birth cohort-specific education tertiles, alcohol use, smoking, BMI, physical inactivity, and ischemic heart disease mortality in sex-stratified Cox PH models. (DOCX)

S14 Table Sensitivity Analysis 4: Natural direct and indirect effects (hazard ratio scale) of educational level based on birth cohort-specific education tertiles. (DOCX)

## Abbreviations

ACA: Affordable Care Act
BMI: body mass index
BRF: behavioral risk factor
CVD: cardiovascular disease
HR: hazard ratio
IHD: ischemic heart disease
NDE: natural direct effect
NDI: National Death Index
NIE: natural indirect effect
PAF: population attributable fraction
PH: proportional hazard
SES: socioeconomic status
TE: total effect
UHC: universal health coverage
US: United States
