# Supplementary material for: Behavioral risk factors and socioeconomic inequalities in ischemic heart disease mortality in the United States: A causal mediation analysis using record linkage data
Source: PLoS Med. 2024 Sep 17;21(9):e1004455. doi: 10.1371/journal.pmed.1004455 (PMC11407680; doi:10.1371/journal.pmed.1004455)
Supplement: S1 Analysis Plan — (DOCX) [file pmed.1004455.s001.docx]

# **S1 Analysis Plan**

# **Research Questions:**

1. Evaluate the joint effect of health behaviors (namely alcohol use, smoking, BMI, and physical activity) with SES (educational attainment) on ischemic heart disease (IHD) mortality.
2. Evaluate the extent to which the association between SES with IHD mortality is mediated by health behaviors.

**Hypotheses:**

1. The harmful associations between health behaviors and IHD mortality would be stronger among people with low SES compared with those with high SES. The protective association of light-to-moderate alcohol use with IHD mortality would be more pronounced among people with high SES than those with low SES.
2. Health behaviors partially explain the association between SES and IHD mortality.

# **Data Requirements:**

1. Survey, Years, Files:

NHIS 1997-2018 Household, Family, Person, and Sample Adult Files.

Restricted-use Linked Mortality File 2019.

1. Restricted-use Data:

IHD mortality is defined based on the ICD-10 codes I20-I25. A small minority of participants in NHIS 1997-1998 will have cause of death data based on ICD-9. These ICD-9 codes will be translated into ICD-10.

The main outcome of interest is time from the NHIS survey to death by IHD, last presumed alive, or censored. The age at NHIS survey is also a key covariate of interest. To this end, the date of birth, date of NHIS interview, and date of death or last follow-up will be required to calculate age and follow-up. The ages at each timepoint that are available in the mortality data will also be retrieved. The cause of death (using ICD-10 codes) is required, as outlined in the table above.

The specific restricted-access variables required are:

1. NHIS restricted data 1997-2018:

| Variable | Description | Reason/use of variable |
| --- | --- | --- |
| AGE | Age | The public use version of this variable does not provide the exact age for those older than 85 years of age. |

1. Mortality file restricted data 2019:

| Variable | Description | Reason/use of variable |
| --- | --- | --- |
| PUBLICID | Participant ID | To link data to NHIS |
| SA_WGT_NEW | Weight Adjusted for Ineligible Respondents | To adjust analyses using sampling weights |
| MORTSTAT | Final mortality status | To identify death from any cause |
| DOBDAY | DOBDAY | To calculate the exact age at NHIS interview, age at death, and follow-up time |
| DOBMONTH | DOBMONTH |  |
| DOBYEAR | DOBYEAR |  |
| DODDAY | DODDAY |  |
| DODMONTH | DODMONTH |  |
| DODYEAR | DODYEAR |  |
| INTVDAY | INTVDAY |  |
| INTVMONTH | INTVMONTH |  |
| INTVYEAR | INTVYEAR |  |
| AGEINTV | Age at NHIS Interview | To verify the calculation of the age at NHIS survey, death, and follow-up time |
| AGEDEATH | Age at Death |  |
| AGEPRALV | Age When Last Presumed Alive |  |
| ICD_10REV | ICD-10 Underlying Cause of Death (available 1999 forward) | To identify the cause of death |
| ICD_9REV | ICD-9 Underlying Cause of Death (available up to 1998) | To identify the cause of death |

Notably, once follow-up times and ages are calculated, the dates will be removed the data.

1. Merge Variables:

PUBLICID will be used to merge the public and restricted NCHS data files.

# **Methodology:**

1. Unit or Level of Analysis and Subpopulation(s):

Unit of analysis – individual

Subpopulation – adults, aged 18 years or older*

* The minimum age may be older for specific analyses; for example, analyses with educational attainment as the primary exposure variable would utilize an older age (e.g., 25 or 30 years) on the basis that younger individuals would not have yet achieved their final education attainment at the time of the NHIS.

1. Analysis Plan:

All analyses will be completed in R using the following packages (for data manipulation and analysis): *haven, tidyverse, janitor, skimr, gmodels, tableone, survival, survminer, timereg, survey, biostat3, VGAM, tidycmprsk.* The operationalization of each variable has been listed at the end of this section.

**Objective 1**

Descriptive statistics will include the frequency and proportion of IHD deaths and the mortality rate per 100,000 person years. The joint effect of SES (operationalized as educational attainment) with health behaviors (alcohol use, smoking, BMI, physical activity) will be evaluated with hazard models on the multiplicative scale (using i) Cox proportional hazards models with the *survey* package, and ii) Fine-Gray sub-distribution hazard models with the *tidycmprsk* package to account for competing risks) and additive scale (using Aalen’s additive hazards models with the *timereg* package). To evaluate additive interaction from the hazard models, the relative excess risk due to interaction (RERI) will be calculated. Each health behavior will be evaluated one at a time, and models will be adjusted for age (used as the time scale), race/ethnicity, marital status, and survey year. Separate models will be fit for male and female.

**Objective 2**

The extent to which the relationship between SES (operationalized as educational attainment) on IHD mortality is mediated by health behaviors will be evaluated using causal mediation, fitting an additive hazard model with the marginal structural approach described by Lange et al. [1]. Using the *VGAM* and *timereg* package, the model will include multiple mediators (alcohol use, smoking, BMI, and physical activity) and covariates (age as the time-scale, marital status, race/ethnicity, survey year). Separate models will be fit for male and female participants.

**Operationalization of variables:**

The coding/categorization of variables will be as follows:

- Mortality 🡪 time to death, censored, or last assumed alive. Analyses will be repeated for each cause of death.
- Education 🡪 low (high school diploma or less); medium (some college but no bachelor’s degree); high (bachelor’s degree or more).
- Race/ethnicity 🡪 non-Hispanic White; non-Hispanic Black; Hispanic; other.
- Alcohol use 🡪 grams of alcohol per day categorized as never drinkers, former drinkers, category I, category II, category III drinkers.
- Smoking 🡪 never smoker; former smoker; current some day smoker; current everyday smoker.
- BMI 🡪 underweight; healthy weight; overweight; obese.
- Physical activity 🡪 sedentary; somewhat active; active.
- Age 🡪 continuous.
- Marital status 🡪 married/cohabitating; never married, widowed, divorced, or separated.
- Sex 🡪 male; female.

1. **Complex Survey Design:**

The Cox proportional hazards models will account for the sample weights and design variables. The analysis will be conducted using the *svycoxph* command from the *survey* package. The methodology outlined in the Variance Estimation Guidance and each NHIS Survey description will be followed to create the sample weights and design variables (e.g. sample weights were divided by 18 given that 18 years of NHIS data were pooled; PSU variables were merged; 1000 or 2000 was added to the stratum variables prior to merging, and that the survey package in R was used).

The *timereg* package (used for the additive hazard models and causal mediation) and tidycmprsk package (used for sub-distribution hazard models) are not able to incorporate sample weights nor design variables.

**Reference**

1. Lange T, Rasmussen M, Thygesen LC. Assessing natural direct and indirect effects through multiple pathways. Am J Epidemiol. 2014;179(4):513-8. doi: 10.1093/aje/kwt270.
