# Supplementary material for: Behavioral risk factors and socioeconomic inequalities in ischemic heart disease mortality in the United States: A causal mediation analysis using record linkage data
Source: PLoS Med. 2024 Sep 17;21(9):e1004455. doi: 10.1371/journal.pmed.1004455 (PMC11407680; doi:10.1371/journal.pmed.1004455)
Supplement: S1 Methods — (DOCX) [file pmed.1004455.s002.docx]

**S1 Methods. Description of Differences Between the Analysis Plan and the Study Performed.**

The study addresses one of the several research questions in a larger project. S1 Analysis Plan describes the research questions (“*Evaluate the extent to which the association between SES with IHD mortality is mediated by health behaviors”*), variables, and methodology for this study.

Our analyses mostly adhere to the original analysis plan with some minor changes to overcome the limitations of the analysis plan.

1. We slightly modified the categories of alcohol use because male and female participants have substantially different ranges of alcohol intake and there were very few females drinking >40 g/day, we combined all females who drank >20 g/day, thus ending up with different number of categories for males and females, i.e., 6 groups for male and 4 groups for female, respectively: 1) Lifetime abstainer (never drank alcohol in the past 12 months and never had 12+ drinks in any one year, reference group), 2) Former drinker (never drank alcohol in the past 12 months but ever had 12+ drinks in any one year), 3) Category I (past year daily average of (0, 20] g for both female and male), Category II (past year daily average of (20, 40] g for male and >20 g for female), Category III (past-year daily average of (40, 60] g for male only), and Category IV (past-year daily average >60 g for male only).
2. Educational groups based on birth cohort-specific tertiles were added in response to a reviewer comment.
3. According to the S1 Analysis Plan, Cox proportional hazards (PH) models were applied to evaluate potential interactions of SES with alcohol use, smoking, BMI, and physical activity on IHD mortality. The NHIS sample is selected by a multistage process, beginning with selection of geographic areas called primary sampling units (PSU) that are defined within sampling strata [2]. It is recommended that complex survey design should be accounted for in statistical analyses using NHIS data [3]. Cox PH models can account for the survey weights, strata, and PSUs of NHIS, deriving unbiased effect estimates and asymptotically unbiased variance estimation [4]. This also makes our results comparable to the literature on the same topic in other countries [5], as Cox model is the mostly used technique for survival analysis. Aalen’s additive hazard model was not used because it could not possibly account for the complex survey design of NHIS data [6].
4. To keep all statistical analyses consistent and comparable [4, 5], we again applied the Cox models (rather than Aalen’s models [6, 7]) for causal mediation analysis to evaluate the extent to which the relationship between SES and IHD mortality can be explained by behavioral risk factors [1, 8]. The interpretation of causal mediation analysis with marginal structural model on the relative risk scale has been extensively documented in previous literature [9-11].
5. As described in the S1 Analysis Plan, the *timereg* package (used for the additive hazard models and causal mediation) are not able to incorporate sample weights nor design variables [6], we overcame this limitation by including survey weights in our final analysis using Cox models as the marginal structural model in this manuscript according to the weight adjustment procedures described in Vart et al. (2015) [10], by multiplying the mediation weights by the survey sampling-weights.

**References**

1. Lange T, Rasmussen M, Thygesen LC. Assessing natural direct and indirect effects through multiple pathways. Am J Epidemiol. 2014;179(4):513-8. doi: 10.1093/aje/kwt270.

2. Supplementary Resources for National Health Interview Survey Public Use Files With Variance Estimation Singleton PSUs [Internet]. 2015. Available from: <https://www.cdc.gov/nchs/nhis/singleton_psu.htm>. Accessed July 18, 2024.

3. Variance Estimation Guidance, NHIS 2016-2017 (Adapted from NHIS Survey Description Documents) [Internet]. 2018. Available from: <https://www.cdc.gov/nchs/data/nhis/2016var.pdf>. Accessed July 18, 2024.

4. Zhu Y, Llamosas-Falcón L, Kerr W, Puka K, Probst C. Differential Associations of Alcohol Use With Ischemic Heart Disease Mortality by Socioeconomic Status in the US, 1997-2018. JAMA Netw Open. 2024;7(2):e2354270. doi: 10.1001/jamanetworkopen.2023.54270.

5. Degerud E, Ariansen I, Ystrom E, Graff-Iversen S, Høiseth G, Mørland J, et al. Life course socioeconomic position, alcohol drinking patterns in midlife, and cardiovascular mortality: Analysis of Norwegian population-based health surveys. PLoS Med. 2018;15(1):e1002476. doi: 10.1371/journal.pmed.1002476.

6. Puka K, Buckley C, Mulia N, Lasserre AM, Rehm J, Probst C. Educational attainment and lifestyle risk factors associated with all-cause mortality in the US. JAMA Health Forum. 2022;3(4):e220401. doi: 10.1001/jamahealthforum.2022.0401.

7. Peña S, Mäkelä P, Laatikainen T, Härkänen T, Männistö S, Heliövaara M, et al. Joint effects of alcohol use, smoking and body mass index as an explanation for the alcohol harm paradox: causal mediation analysis of eight cohort studies. Addiction. 2021;116(8):2220-30.

8. Lange T, Vansteelandt S, Bekaert M. A simple unified approach for estimating natural direct and indirect effects. Am J Epidemiol. 2012;176(3):190-5. doi: 10.1093/aje/kwr525.

9. VanderWeele TJ, Vansteelandt S. Odds ratios for mediation analysis for a dichotomous outcome. Am J Epidemiol. 2010;172(12):1339-48. doi: 10.1093/aje/kwq332.

10. Vart P, Gansevoort RT, Crews DC, Reijneveld SA, Bültmann U. Mediators of the association between low socioeconomic status and chronic kidney disease in the United States. Am J Epidemiol. 2015;181(6):385-96. doi: 10.1093/aje/kwu316.

11. Menvielle G, Franck J, Radoi L, Sanchez M, Fevotte J, Guizard A, et al. Quantifying the mediating effects of smoking and occupational exposures in the relation between education and lung cancer: the ICARE study. Eur J Epidemiol. 2016;31(12):1213-21. doi: <https://doi.org/10.1007/s10654-016-0182-2>.
