# Supplementary material for: Behavioral risk factors and socioeconomic inequalities in ischemic heart disease mortality in the United States: A causal mediation analysis using record linkage data
Source: PLoS Med. 2024 Sep 17;21(9):e1004455. doi: 10.1371/journal.pmed.1004455 (PMC11407680; doi:10.1371/journal.pmed.1004455)
Supplement: S1 RECORD Statement — (DOCX) [file pmed.1004455.s003.docx]

**S1 The RECORD statement – checklist of items, extended from the STROBE statement, that should be reported in observational studies using routinely collected health data.**

|  | **Item No.** | **STROBE items** | **Location in manuscript where items are reported** | **RECORD items** | **Location in manuscript where items are reported** |
| --- | --- | --- | --- | --- | --- |
| **Title and abstract** | | | | | |
|  | 1 | (a) Indicate the study’s design with a commonly used term in the title or the abstract (b) Provide in the abstract an informative and balanced summary of what was done and what was found | (a) The study design was indicated in the title (“Record Linkage”) and **Abstract** (“data from the 1997-2018 National Health Interview Survey with mortality follow-up until 12/31/2019 from the National Death Index”.  (b) Summary of methods and findings has been provided in the **Abstract**. | RECORD 1.1: The type of data used should be specified in the title or abstract. When possible, the name of the databases used should be included.  RECORD 1.2: If applicable, the geographic region and timeframe within which the study took place should be reported in the title or abstract.  RECORD 1.3: If linkage between databases was conducted for the study, this should be clearly stated in the title or abstract. | 1.1 The type and name of data used has been specified in the **Abstract**.  1.2 The geographic region (“United States”) has been reported in the title and time frame (data from 1997-2018 with mortality follow-up until 12/31/2019) has been mentioned in the **Abstract**.  1.3 Linkage was conducted and stated clearly in the title. |
| **Introduction** | | | | | |
| Background rationale | 2 | Explain the scientific background and rationale for the investigation being reported | The scientific background and rationale for the investigation has been explained in the **Introduction**. |  |  |
| Objectives | 3 | State specific objectives, including any prespecified hypotheses | Specific objectives and hypotheses were stated in the final paragraph of the **Introduction**. |  |  |
| **Methods** | | | | | |
| Study Design | 4 | Present key elements of study design early in the paper | The Record Linkage study design and key elements were presented in the first 2 paragraphs of *Data source and measures* in **Methods**. |  |  |
| Setting | 5 | Describe the setting, locations, and relevant dates, including periods of recruitment, exposure, follow-up, and data collection | *Data source and measures* in **Methods**. |  |  |
| Participants | 6 | *(a) Cohort study* - Give the eligibility criteria, and the sources and methods of selection of participants. Describe methods of follow-up  *Case-control study* - Give the eligibility criteria, and the sources and methods of case ascertainment and control selection. Give the rationale for the choice of cases and controls  *Cross-sectional study* - Give the eligibility criteria, and the sources and methods of selection of participants  *(b) Cohort study* - For matched studies, give matching criteria and number of exposed and unexposed  *Case-control study* - For matched studies, give matching criteria and the number of controls per case | Cohort study – data linkage and follow-up were described in *Data source and measures* in **Methods**. | RECORD 6.1: The methods of study population selection (such as codes or algorithms used to identify subjects) should be listed in detail. If this is not possible, an explanation should be provided.  RECORD 6.2: Any validation studies of the codes or algorithms used to select the population should be referenced. If validation was conducted for this study and not published elsewhere, detailed methods and results should be provided.  RECORD 6.3: If the study involved linkage of databases, consider use of a flow diagram or other graphical display to demonstrate the data linkage process, including the number of individuals with linked data at each stage. | Described in S1 Analysis Plan.  Data linkage was performed by the NCHS staff and was described in *Data source and measures* in **Methods**. |
| Variables | 7 | Clearly define all outcomes, exposures, predictors, potential confounders, and effect modifiers. Give diagnostic criteria, if applicable. | Defined in *Data source and measures* in **Methods**. | RECORD 7.1: A complete list of codes and algorithms used to classify exposures, outcomes, confounders, and effect modifiers should be provided. If these cannot be reported, an explanation should be provided. | Detailed descriptions of the coding of exposures, outcomes, mediators, and confounders have been provided in *Data source and measures* in **Methods** as well as S1 Protocol. |
| Data sources/ measurement | 8 | For each variable of interest, give sources of data and details of methods of assessment (measurement).  Describe comparability of assessment methods if there is more than one group | *Data source and measures* in **Methods** and S1 Analysis Plan. |  |  |
| Bias | 9 | Describe any efforts to address potential sources of bias | Sensitivity analyses described in *Statistical analysis* in **Methods**. |  |  |
| Study size | 10 | Explain how the study size was arrived at | *Data source and measures* in **Methods**; First paragraph of Results |  |  |
| Quantitative variables | 11 | Explain how quantitative variables were handled in the analyses. If applicable, describe which groupings were chosen, and why | 3rd paragraph of *Data source and measures* in **Methods**. |  |  |
| Statistical methods | 12 | (a) Describe all statistical methods, including those used to control for confounding  (b) Describe any methods used to examine subgroups and interactions  (c) Explain how missing data were addressed  (d) *Cohort study* - If applicable, explain how loss to follow-up was addressed  *Case-control study* - If applicable, explain how matching of cases and controls was addressed  *Cross-sectional study* - If applicable, describe analytical methods taking account of sampling strategy  (e) Describe any sensitivity analyses | (a) *Statistical analysis* in **Methods**.  (b) 1st paragraph of *Statistical analysis* in **Methods**.  (c) List-wise deletion (only a small proportion of missing data), see the 2nd sentence in Results.  (e) Sensitivity analyses were described in the final paragraph of *Statistical analysis*. |  |  |
| Data access and cleaning methods |  | .. |  | RECORD 12.1: Authors should describe the extent to which the investigators had access to the database population used to create the study population.  RECORD 12.2: Authors should provide information on the data cleaning methods used in the study. | 12.1 *Data source and measures* in **Methods**. Data were for restricted use only at the NCHS Research Data Center.  12.2 Descriptions of data linkage were provided in *Data source and measures* in **Methods**. Listwise deletion was used for missing data (first paragraph of **Results**). |
| Linkage |  | .. |  | RECORD 12.3: State whether the study included person-level, institutional-level, or other data linkage across two or more databases. The methods of linkage and methods of linkage quality evaluation should be provided. | Stated in *Data source and measures*, individual-level data linkage based on both deterministic and probabilistic approaches was performed. |
| **Results** | | | | | |
| Participants | 13 | (a) Report the numbers of individuals at each stage of the study (*e.g.*, numbers potentially eligible, examined for eligibility, confirmed eligible, included in the study, completing follow-up, and analysed)  (b) Give reasons for non-participation at each stage.  (c) Consider use of a flow diagram | Eligibility was described in *Data source and measures*. | RECORD 13.1: Describe in detail the selection of the persons included in the study (*i.e.,* study population selection) including filtering based on data quality, data availability and linkage. The selection of included persons can be described in the text and/or by means of the study flow diagram. | *Data source and measures*. |
| Descriptive data | 14 | (a) Give characteristics of study participants (*e.g.*, demographic, clinical, social) and information on exposures and potential confounders  (b) Indicate the number of participants with missing data for each variable of interest  (c) *Cohort study* - summarise follow-up time (*e.g.*, average and total amount) | (a) Characteristics of study participants and information on exposures and confounders have been described in Table 1.  (b) Number of participants with missing data for each variable of interest has been provided in Zhu et al. (2024), eTable 1 as we cited in the first paragraph of Results.  (c) Average follow-up time and total person-years were summarized in the first sentence of **Results** and Table 1. |  |  |
| Outcome data | 15 | *Cohort study* - Report numbers of outcome events or summary measures over time  *Case-control study* - Report numbers in each exposure category, or summary measures of exposure  *Cross-sectional study* - Report numbers of outcome events or summary measures | Cohort study – the number of outcome events (IHD mortality) was reported in the first paragraph of **Results**. |  |  |
| Main results | 16 | (a) Give unadjusted estimates and, if applicable, confounder-adjusted estimates and their precision (e.g., 95% confidence interval). Make clear which confounders were adjusted for and why they were included  (b) Report category boundaries when continuous variables were categorized  (c) If relevant, consider translating estimates of relative risk into absolute risk for a meaningful time period | (a) We presented both minimally adjusted estimates and fully adjusted estimates with 95% CIs in Table 2. Confounders adjusted for were explained explicitly in the table and footnote as well as the last paragraph under *Data source and measures*.  (b) Category boundaries were reported in the 4th paragraph under *Data sources and measures*.  (c) We did not translate estimates of relative risk into absolute risk, the justification has been provided in S1 Methods. |  |  |
| Other analyses | 17 | Report other analyses done—e.g., analyses of subgroups and interactions, and sensitivity analyses | We discussed 4 sensitivity analyses done in the last 4 paragraphs of **Results**. |  |  |
| **Discussion** | | | | | |
| Key results | 18 | Summarise key results with reference to study objectives | We have summarized our key findings in the first paragraph of **Discussion**. |  |  |
| Limitations | 19 | Discuss limitations of the study, taking into account sources of potential bias or imprecision. Discuss both direction and magnitude of any potential bias | We have extensively discussed the limitations of this study in **Discussion** (under the subtitle of *Limitations*), and how they might have biased our results. | RECORD 19.1: Discuss the implications of using data that were not created or collected to answer the specific research question(s). Include discussion of misclassification bias, unmeasured confounding, missing data, and changing eligibility over time, as they pertain to the study being reported. | Misclassification bias and unmeasured confounding have been extensively discussed in the *Limitations* section. Missing data only accounted for a very small portion of the data thus were not discussed as a limitation. |
| Interpretation | 20 | Give a cautious overall interpretation of results considering objectives, limitations, multiplicity of analyses, results from similar studies, and other relevant evidence | Conclusions paragraph. |  |  |
| Generalisability | 21 | Discuss the generalisability (external validity) of the study results | Generalisability was discussed in the *Strength* section of **Discussion**. |  |  |
| **Other Information** | | | | | |
| Funding | 22 | Give the source of funding and the role of the funders for the present study and, if applicable, for the original study on which the present article is based | Funding source was provided in our submission. |  |  |
| Accessibility of protocol, raw data, and programming code |  | .. |  | RECORD 22.1: Authors should provide information on how to access any supplemental information such as the study protocol, raw data, or programming code. | Supplementary information have been provided and cited in the manuscript. |

*Reference: Benchimol EI, Smeeth L, Guttmann A, Harron K, Moher D, Petersen I, Sørensen HT, von Elm E, Langan SM, the RECORD Working Committee. The REporting of studies Conducted using Observational Routinely-collected health Data (RECORD) Statement. *PLoS Medicine* 2015;12(10):e1001885. doi: 10.1371/journal.pmed.1001885.

*Checklist is protected under Creative Commons Attribution ([CC BY](http://creativecommons.org/licenses/by/4.0/)) license.
