# Supplementary material for: Behavioral risk factors and socioeconomic inequalities in ischemic heart disease mortality in the United States: A causal mediation analysis using record linkage data
Source: PLoS Med. 2024 Sep 17;21(9):e1004455. doi: 10.1371/journal.pmed.1004455 (PMC11407680; doi:10.1371/journal.pmed.1004455)
Supplement: S1 Fig — (DOCX) [file pmed.1004455.s005.docx]

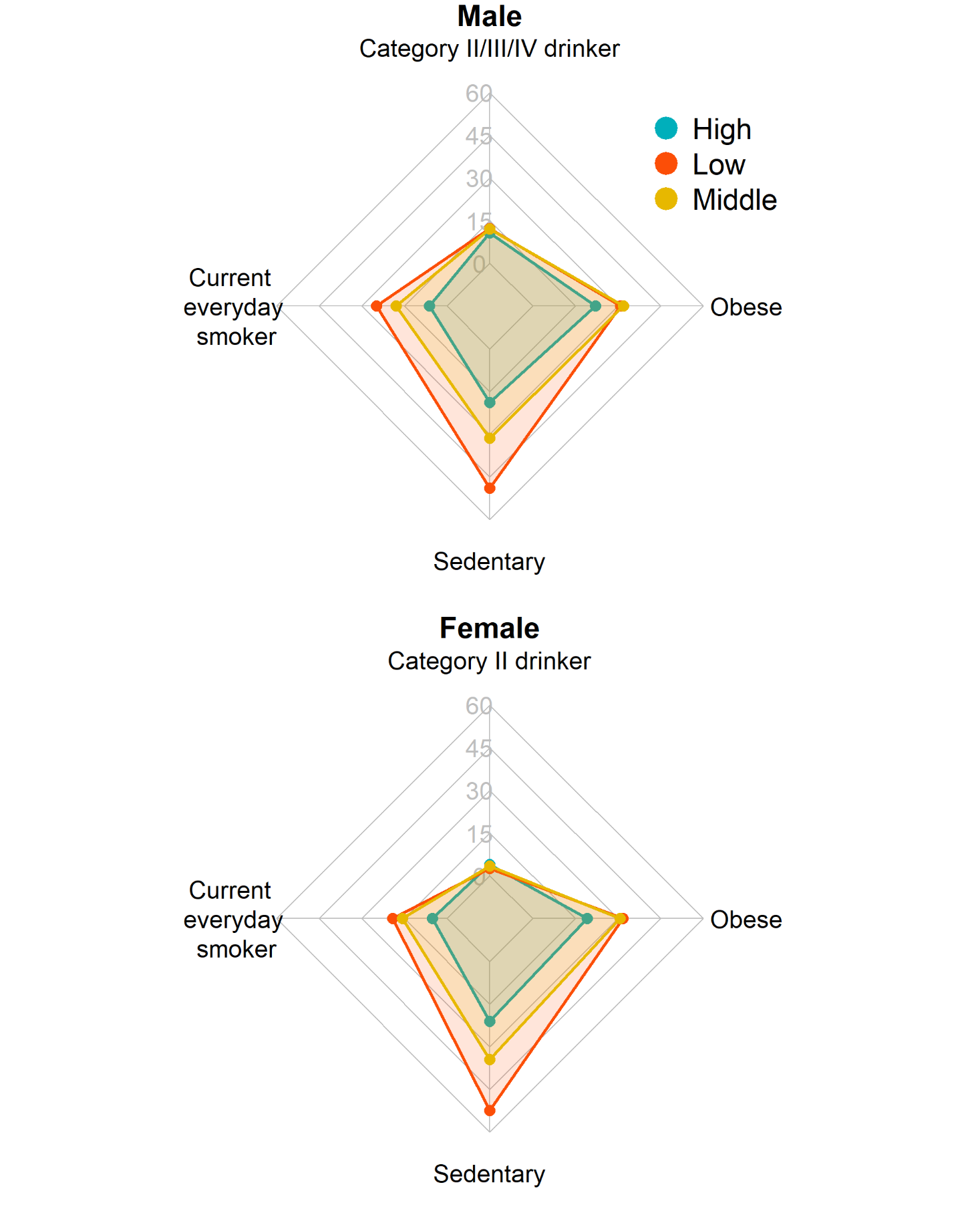


**S1 Fig.** Sampling-weighted Prevalence of Behavioral Risk Factors by Sex and Education. Alcohol Use Category II: (20, 40] g/day for male and >20 g for female; Category III: (40, 60] g/day for male only; Category IV: >60 g/day for male only.
