# Supplementary material for: Behavioral risk factors and socioeconomic inequalities in ischemic heart disease mortality in the United States: A causal mediation analysis using record linkage data
Source: PLoS Med. 2024 Sep 17;21(9):e1004455. doi: 10.1371/journal.pmed.1004455 (PMC11407680; doi:10.1371/journal.pmed.1004455)
Supplement: S1 Table — (DOCX) [file pmed.1004455.s006.docx]

**S1 Table.** Hazard Ratios from Cox Proportional Hazards Model with Interaction between Education and Sex.

|  | HR | 95% CI | p-value |
| --- | --- | --- | --- |
| Education |  |  |  |
| Low | 1.27 | (1.18, 1.37) | <.001 |
| Middle | 1.32 | (1.21, 1.44) | <.001 |
| High | ref |  |  |
| Sex |  |  |  |
| Male | ref |  |  |
| Female | 0.37 | (0.33, 0.42) | <.001 |
| **Interaction between education and sex** |  |  |  |
| Low:Female | 1.28 | (1.13, 1.45) | <.001 |
| Middle:Female | 1.06 | (0.92, 1.23) | 0.428 |
| **Covariates** |  |  |  |
| Marital status |  |  |  |
| Not married/cohabitating | ref |  |  |
| Married/cohabitating | 0.68 | (0.65, 0.71) | <.001 |
| Race/ethnicity |  |  |  |
| White | ref |  |  |
| Black | 0.93 | (0.87, 1) | 0.05 |
| Hispanic | 0.76 | (0.71, 0.82) | <.001 |
| Other | 0.73 | (0.64, 0.84) | <.001 |
| Alcohol use |  |  |  |
| Lifetime abstainer | ref |  |  |
| Former drinker | 1.03 | (0.96, 1.1) | 0.357 |
| Category I: (0, 20] g/day | 0.71 | (0.67, 0.74) | <.001 |
| Category II: (20, 40] g/day for male; >20 g/day for female | 0.7 | (0.63, 0.78) | <.001 |
| Category III: (40, 60] g/day for male only | 0.9 | (0.75, 1.08) | 0.252 |
| Category IV: >60 g/day for male only | 1.08 | (0.88, 1.32) | 0.48 |
| Smoking status |  |  |  |
| Never smoker | ref |  |  |
| Former smoker | 1.4 | (1.33, 1.47) | <.001 |
| Current someday smoker | 1.82 | (1.6, 2.07) | <.001 |
| Current everyday smoker | 2.37 | (2.23, 2.53) | <.001 |
| BMI |  |  |  |
| Underweight | 1.37 | (1.18, 1.59) | <.001 |
| Healthy weight | ref |  |  |
| Overweight | 1 | (0.95, 1.05) | 0.914 |
| Obese | 1.35 | (1.28, 1.43) | <.001 |
| Physical inactivity |  |  |  |
| Active | ref |  |  |
| Somewhat active | 1.28 | (1.2, 1.37) | <.001 |
| Sedentary | 1.68 | (1.6, 1.77) | <.001 |
