# Supplementary material for: Behavioral risk factors and socioeconomic inequalities in ischemic heart disease mortality in the United States: A causal mediation analysis using record linkage data
Source: PLoS Med. 2024 Sep 17;21(9):e1004455. doi: 10.1371/journal.pmed.1004455 (PMC11407680; doi:10.1371/journal.pmed.1004455)
Supplement: S2 Table — (DOCX) [file pmed.1004455.s007.docx]

**S2 Table.** Tests of Proportional Hazards Assumptions for Cox Proportional Hazards Models in Table 2.

|  | Male | | | | | | Female | | | | | |
| --- | --- | --- | --- | --- | --- | --- | --- | --- | --- | --- | --- | --- |
|  | Minimally adjusted | | | Fully adjusted | | | Minimally adjusted | | | Fully adjusted | | |
|  | Chi-square statistics | Degrees of freedom | p-value | Chi-square statistics | Degrees of freedom | p-value | Chi-square statistics | Degrees of freedom | p-value | Chi-square statistics | Degrees of freedom | p-value |
| *Educational level for main analysis* | | | | | | | | | | | | |
| Education | 0.00041 | 2 | 1 | 0.000229 | 2 | 1 | 0.000312 | 2 | 1 | 0.00018 | 2 | 1 |
| Marital status | 0.000238 | 1 | 0.99 | 0.000211 | 1 | 0.99 | 0.000165 | 1 | 0.99 | 0.000128 | 1 | 0.99 |
| Race and ethnicity | 0.000151 | 3 | 1 | 0.000205 | 3 | 1 | 0.000151 | 3 | 1 | 0.000156 | 3 | 1 |
| Alcohol use | 0.000146 | 21 | 1 | 0.000186 | 5 | 1 | 5.52E-05 | 21 | 1 | 0.000194 | 3 | 1 |
| Smoking | 0.000858 | 27 | 1 | 0.00024 | 3 | 1 | 0.000625 | 27 | 1 | 0.000147 | 3 | 1 |
| BMI |  |  |  | 9.16E-05 | 3 | 1 |  |  |  | 0.000161 | 3 | 1 |
| Physical inactivity |  |  |  | 8.25E-05 | 2 | 1 |  |  |  | 0.000242 | 2 | 1 |
| Survey year |  |  |  | 0.000186 | 21 | 1 |  |  |  | 5.32E-05 | 21 | 1 |
| Global test |  |  |  | 0.001126 | 40 | 1 |  |  |  | 0.001004 | 38 | 1 |
