# Supplementary material for: Behavioral risk factors and socioeconomic inequalities in ischemic heart disease mortality in the United States: A causal mediation analysis using record linkage data
Source: PLoS Med. 2024 Sep 17;21(9):e1004455. doi: 10.1371/journal.pmed.1004455 (PMC11407680; doi:10.1371/journal.pmed.1004455)
Supplement: S3 Table — (DOCX) [file pmed.1004455.s008.docx]

**S3 Table.** Interaction Effects between Education and Alcohol Use on Ischemic Heart Disease Mortality by Sex.

|  | **Male** | | | **Female** | | |
| --- | --- | --- | --- | --- | --- | --- |
|  | HR | 95% CI | p-value | HR | 95% CI | p-value |
| **Main effects** |  |  |  |  |  |  |
| Education |  |  |  |  |  |  |
| Low | 1.18 | (1.02, 1.36) | 0.03 | 1.34 | (1.14, 1.57) | <.001 |
| Middle | 1.37 | (1.15, 1.64) | 0.001 | 1.23 | (1.04, 1.47) | 0.02 |
| High | ref |  |  | ref |  |  |
| Alcohol use |  |  |  |  |  |  |
| Lifetime abstainer | ref |  |  | ref |  |  |
| Former drinker | 1.06 | (0.85, 1.34) | 0.59 | 1.13 | (0.79, 1.6) | 0.51 |
| Category I: (0, 20] g/day | 0.66 | (0.57, 0.77) | <.001 | 0.5 | (0.41, 0.62) | <.001 |
| Category II: (20, 40] g/day for male; >20 g/day for female | 0.7 | (0.54, 0.9) | 0.005 | 0.58 | (0.34, 0.96) | 0.04 |
| Category III: (40, 60] g/day for male only | 0.97 | (0.65, 1.44) | 0.88 | - |  |  |
| Category IV: >60 g/day for male only | 0.86 | (0.51, 1.44) | 0.56 | - |  |  |
| Smoking |  |  |  |  |  |  |
| Never smoker | ref |  |  | ref |  |  |
| Former smoker | 1.42 | (1.32, 1.52) | <.001 | 1.43 | (1.33, 1.53) | <.001 |
| Current someday smoker | 1.75 | (1.48, 2.06) | <.001 | 1.98 | (1.62, 2.43) | <.001 |
| Current everyday smoker | 2.42 | (2.22, 2.64) | <.001 | 2.32 | (2.1, 2.56) | <.001 |
| BMI |  |  |  |  |  |  |
| Underweight | 1.51 | (1.16, 1.96) | 0.002 | 1.29 | (1.06, 1.56) | 0.009 |
| Healthy weight | ref |  |  | ref |  |  |
| Overweight | 0.97 | (0.9, 1.04) | 0.43 | 1.02 | (0.95, 1.1) | 0.56 |
| Obese | 1.36 | (1.26, 1.47) | <.001 | 1.29 | (1.2, 1.39) | <.001 |
| Physical inactivity |  |  |  |  |  |  |
| Active | ref |  |  | ref |  |  |
| Somewhat active | 1.28 | (1.17, 1.39) | <.001 | 1.32 | (1.18, 1.47) | <.001 |
| Sedentary | 1.6 | (1.5, 1.71) | <.001 | 1.82 | (1.67, 1.99) | <.001 |
| **Interaction between education and alcohol use** |  |  |  |  |  |  |
| Low:Former drinker | 1.02 | (0.79, 1.33) | 0.85 | 1 | (0.69, 1.45) | 0.99 |
| Middle:Former drinker | 0.84 | (0.62, 1.14) | 0.25 | 0.76 | (0.5, 1.17) | 0.21 |
| Low:Category I | 1.22 | (1.02, 1.45) | 0.03 | 1.35 | (1.09, 1.67) | 0.006 |
| Middle:Category I | 1.01 | (0.82, 1.25) | 0.92 | 1.35 | (1.06, 1.72) | 0.02 |
| Low:Category II | 1.22 | (0.9, 1.66) | 0.21 | 1 | (0.54, 1.85) | 0.99 |
| Middle:Category II | 0.81 | (0.57, 1.15) | 0.23 | 1.14 | (0.6, 2.2) | 0.68 |
| Low:Category III | 0.83 | (0.51, 1.35) | 0.44 |  |  |  |
| Middle:Category III | 1.04 | (0.62, 1.75) | 0.89 |  |  |  |
| Low:Category IV | 1.45 | (0.82, 2.58) | 0.20 |  |  |  |
| Middle:Category IV | 0.89 | (0.49, 1.62) | 0.71 |  |  |  |

Note: This model adjusted for marital status, race and ethnicity, and categorical survey year.
