# Supplementary material for: Behavioral risk factors and socioeconomic inequalities in ischemic heart disease mortality in the United States: A causal mediation analysis using record linkage data
Source: PLoS Med. 2024 Sep 17;21(9):e1004455. doi: 10.1371/journal.pmed.1004455 (PMC11407680; doi:10.1371/journal.pmed.1004455)
Supplement: S4 Table — (DOCX) [file pmed.1004455.s009.docx]

**S4 Table.** Interaction Effects between Education and Smoking on Ischemic Heart Disease Mortality by Sex.

|  | **Male** | | | **Female** |  |  |
| --- | --- | --- | --- | --- | --- | --- |
|  | HR | 95% CI | p-value | HR | 95% CI | p-value |
| **Main effects** |  |  |  |  |  |  |
| Education |  |  |  |  |  |  |
| Low | 1.27 | (1.12, 1.45) | <.001 | 1.4 | (1.22, 1.61) | <.001 |
| Middle | 1.25 | (1.08, 1.46) | 0.003 | 1.23 | (1.05, 1.44) | 0.01 |
| High | ref |  |  | ref |  |  |
| Smoking |  |  |  |  |  |  |
| Never smoker | ref |  |  | ref |  |  |
| Former smoker | 1.3 | (1.13, 1.5) | <.001 | 1.13 | (0.91, 1.41) | 0.276 |
| Current someday smoker | 1.64 | (1.09, 2.48) | 0.018 | 1.89 | (1, 3.56) | 0.048 |
| Current everyday smoker | 2.64 | (2.12, 3.3) | <.001 | 2.04 | (1.42, 2.93) | <.001 |
| Alcohol use |  |  |  |  |  |  |
| Lifetime abstainer | ref |  |  | ref |  |  |
| Former drinker | 1.04 | (0.95, 1.13) | 0.372 | 1.07 | (0.96, 1.19) | 0.225 |
| Category I: (0, 20] g/day | 0.74 | (0.7, 0.8) | <.001 | 0.66 | (0.61, 0.71) | <.001 |
| Category II: (20, 40] g/day for male; >20 g/day for female | 0.74 | (0.65, 0.84) | <.001 | 0.62 | (0.49, 0.78) | <.001 |
| Category III: (40, 60] g/day for male only | 0.91 | (0.76, 1.1) | 0.327 | - |  |  |
| Category IV: >60 g/day for male only | 1.08 | (0.87, 1.32) | 0.489 | - |  |  |
| BMI |  |  |  |  |  |  |
| Underweight | 1.51 | (1.17, 1.96) | 0.002 | 1.29 | (1.07, 1.56) | 0.009 |
| Healthy weight | ref |  |  | ref |  |  |
| Overweight | 0.97 | (0.9, 1.04) | 0.422 | 1.02 | (0.95, 1.1) | 0.548 |
| Obese | 1.36 | (1.26, 1.48) | <.001 | 1.29 | (1.2, 1.39) | <.001 |
| Physical inactivity |  |  |  |  |  |  |
| Active | ref |  |  | ref |  |  |
| Somewhat active | 1.28 | (1.17, 1.4) | <.001 | 1.32 | (1.18, 1.47) | <.001 |
| Sedentary | 1.6 | (1.5, 1.72) | <.001 | 1.82 | (1.67, 2) | <.001 |
| **Interaction between education and smoking** |  |  |  |  |  |  |
| Low:Former smoker | 1.08 | (0.92, 1.27) | 0.334 | 1.28 | (1, 1.62) | 0.046 |
| Middle:Former smoker | 1.17 | (0.97, 1.42) | 0.099 | 1.34 | (1.03, 1.75) | 0.031 |
| Low:Current someday smoker | 1.12 | (0.7, 1.8) | 0.626 | 1.18 | (0.61, 2.26) | 0.629 |
| Middle:Current someday smoker | 0.99 | (0.58, 1.7) | 0.976 | 0.77 | (0.37, 1.57) | 0.469 |
| Low:Current everyday smoker | 0.92 | (0.72, 1.19) | 0.539 | 1.1 | (0.76, 1.61) | 0.603 |
| Middle:Current everyday smoker | 0.9 | (0.68, 1.18) | 0.434 | 1.32 | (0.89, 1.96) | 0.173 |

Note: This model adjusted for marital status, race and ethnicity, and categorical survey year.
