# Supplementary material for: Behavioral risk factors and socioeconomic inequalities in ischemic heart disease mortality in the United States: A causal mediation analysis using record linkage data
Source: PLoS Med. 2024 Sep 17;21(9):e1004455. doi: 10.1371/journal.pmed.1004455 (PMC11407680; doi:10.1371/journal.pmed.1004455)
Supplement: S5 Table — (DOCX) [file pmed.1004455.s010.docx]

**S5 Table.** Interaction Effects between Education and Physical Inactivity on Ischemic Heart Disease Mortality by Sex.

|  | **Male** |  |  | **Female** |  |  |
| --- | --- | --- | --- | --- | --- | --- |
|  | HR | 95% CI | p-value | HR | 95% CI | p-value |
| **Main effects** |  |  |  |  |  |  |
| Education |  |  |  |  |  |  |
| Low | 1.43 | (1.26, 1.62) | <.001 | 1.86 | (1.52, 2.27) | <.001 |
| Middle | 1.43 | (1.24, 1.64) | <.001 | 1.55 | (1.25, 1.93) | <.001 |
| High | ref |  |  | ref |  |  |
| Smoking |  |  |  |  |  |  |
| Never smoker | ref |  |  | ref |  |  |
| Former smoker | 1.41 | (1.31, 1.52) | <.001 | 1.42 | (1.33, 1.53) | <.001 |
| Current someday smoker | 1.75 | (1.48, 2.07) | <.001 | 1.98 | (1.62, 2.44) | <.001 |
| Current everyday smoker | 2.42 | (2.22, 2.64) | <.001 | 2.32 | (2.1, 2.56) | <.001 |
| Alcohol use |  |  |  |  |  |  |
| Lifetime abstainer | ref |  |  | ref |  |  |
| Former drinker | 1.04 | (0.96, 1.13) | 0.359 | 1.07 | (0.96, 1.19) | 0.223 |
| Category I: (0, 20] g/day | 0.75 | (0.7, 0.8) | <.001 | 0.66 | (0.61, 0.71) | <.001 |
| Category II: (20, 40] g/day for male; >20 g/day for female | 0.74 | (0.65, 0.84) | <.001 | 0.62 | (0.49, 0.77) | <.001 |
| Category III: (40, 60] g/day for male only | 0.91 | (0.76, 1.09) | 0.316 | - |  |  |
| Category IV: >60 g/day for male only | 1.07 | (0.87, 1.32) | 0.5 | - |  |  |
| BMI |  |  |  |  |  |  |
| Underweight | 1.51 | (1.16, 1.96) | 0.002 | 1.29 | (1.06, 1.56) | 0.009 |
| Healthy weight | ref |  |  | ref |  |  |
| Overweight | 0.97 | (0.9, 1.04) | 0.413 | 1.02 | (0.95, 1.1) | 0.56 |
| Obese | 1.36 | (1.26, 1.47) | <.001 | 1.29 | (1.2, 1.39) | <.001 |
| Physical inactivity |  |  |  |  |  |  |
| Active | ref |  |  | ref |  |  |
| Somewhat active | 1.36 | (1.13, 1.63) | 0.001 | 1.59 | (1.21, 2.09) | 0.001 |
| Sedentary | 1.82 | (1.57, 2.11) | <.001 | 2.25 | (1.8, 2.82) | <.001 |
| **Interaction between education and physical inactivity** |  |  |  |  |  |  |
| Low:Sedentary | 0.86 | (0.72, 1.01) | 0.074 | 0.76 | (0.6, 0.98) | 0.033 |
| Middle:Sedentary | 0.84 | (0.69, 1.02) | 0.08 | 0.83 | (0.63, 1.08) | 0.159 |
| Low:Somewhat active | 0.88 | (0.71, 1.1) | 0.26 | 0.76 | (0.56, 1.04) | 0.083 |
| Middle:Somewhat active | 1 | (0.79, 1.28) | 0.979 | 0.89 | (0.63, 1.24) | 0.48 |

Note: This model adjusted for marital status, race and ethnicity, and categorical survey year.
