# Supplementary material for: Behavioral risk factors and socioeconomic inequalities in ischemic heart disease mortality in the United States: A causal mediation analysis using record linkage data
Source: PLoS Med. 2024 Sep 17;21(9):e1004455. doi: 10.1371/journal.pmed.1004455 (PMC11407680; doi:10.1371/journal.pmed.1004455)
Supplement: S6 Table — (DOCX) [file pmed.1004455.s011.docx]

**S6 Table.** Interaction Effects between Education and BMI on Ischemic Heart Disease Mortality by Sex.

|  | **Male** |  |  | **Female** |  |  |
| --- | --- | --- | --- | --- | --- | --- |
|  | HR | 95% CI | p-value | HR | 95% CI | p-value |
| **Main effects** |  |  |  |  |  |  |
| Education |  |  |  |  |  |  |
| Low | 1.41 | (1.23, 1.61) | <.001 | 1.52 | (1.3, 1.77) | <.001 |
| Middle | 1.49 | (1.27, 1.74) | <.001 | 1.27 | (1.06, 1.51) | 0.01 |
| High | ref |  |  | ref |  |  |
| Smoking |  |  |  |  |  |  |
| Never smoker | ref |  |  | ref |  |  |
| Former smoker | 1.41 | (1.31, 1.52) | <.001 | 1.42 | (1.33, 1.53) | <.001 |
| Current someday smoker | 1.75 | (1.48, 2.06) | <.001 | 1.98 | (1.62, 2.44) | <.001 |
| Current everyday smoker | 2.42 | (2.22, 2.64) | <.001 | 2.32 | (2.1, 2.57) | <.001 |
| Alcohol use |  |  |  |  |  |  |
| Lifetime abstainer | ref |  |  | ref |  |  |
| Former drinker | 1.04 | (0.95, 1.13) | 0.365 | 1.07 | (0.96, 1.19) | 0.222 |
| Category I: (0, 20] g/day | 0.74 | (0.7, 0.8) | <.001 | 0.66 | (0.61, 0.71) | <.001 |
| Category II: (20, 40] g/day for male; >20 g/day for female | 0.74 | (0.65, 0.84) | <.001 | 0.62 | (0.49, 0.78) | <.001 |
| Category III: (40, 60] g/day for male only | 0.91 | (0.76, 1.09) | 0.32 | - |  |  |
| Category IV: >60 g/day for male only | 1.07 | (0.87, 1.32) | 0.496 | - |  |  |
| BMI |  |  |  |  |  |  |
| Underweight | 1.62 | (0.89, 2.95) | 0.114 | 1.42 | (0.84, 2.41) | 0.186 |
| Healthy weight | ref |  |  | ref |  |  |
| Overweight | 1.05 | (0.91, 1.21) | 0.53 | 0.92 | (0.72, 1.17) | 0.498 |
| Obese | 1.54 | (1.28, 1.85) | <.001 | 1.31 | (1.02, 1.68) | 0.032 |
| Physical inactivity |  |  |  |  |  |  |
| Active | ref |  |  | ref |  |  |
| Somewhat active | 1.28 | (1.17, 1.4) | <.001 | 1.32 | (1.18, 1.47) | <.001 |
| Sedentary | 1.6 | (1.5, 1.71) | <.001 | 1.83 | (1.67, 2) | <.001 |
| **Interaction between education and BMI** |  |  |  |  |  |  |
| Low:Underweight | 0.91 | (0.46, 1.78) | 0.778 | 0.84 | (0.48, 1.46) | 0.533 |
| Middle:Underweight | 0.97 | (0.38, 2.46) | 0.944 | 1.12 | (0.61, 2.05) | 0.726 |
| Low:Overweight | 0.93 | (0.78, 1.1) | 0.404 | 1.11 | (0.86, 1.43) | 0.418 |
| Middle:Overweight | 0.86 | (0.7, 1.05) | 0.148 | 1.17 | (0.87, 1.58) | 0.298 |
| Low:Obese | 0.88 | (0.71, 1.08) | 0.219 | 0.94 | (0.72, 1.22) | 0.632 |
| Middle:Obese | 0.83 | (0.66, 1.06) | 0.132 | 1.14 | (0.84, 1.55) | 0.386 |

Note: This model adjusted for marital status, race and ethnicity, and categorical survey year.
