# Supplementary material for: Behavioral risk factors and socioeconomic inequalities in ischemic heart disease mortality in the United States: A causal mediation analysis using record linkage data
Source: PLoS Med. 2024 Sep 17;21(9):e1004455. doi: 10.1371/journal.pmed.1004455 (PMC11407680; doi:10.1371/journal.pmed.1004455)
Supplement: S7 Table — (DOCX) [file pmed.1004455.s012.docx]

**S7 Table.** Sensitivity Analysis 1: Causal Mediation Analyses Evaluating One Mediator at a Time.

|  | **Male** |  | **Female** |  |
| --- | --- | --- | --- | --- |
|  | HR (95% CI) | % TE (95% CI) | HR (95% CI) | % TE (95% CI) |
| *Alcohol use* |  |  |  |  |
| *Low education vs high education* |  |  |  |  |
| Natural direct effect | 1.33 (1.22, 1.44) | 85 (79, 89) | 1.51 (1.35, 1.69) | 84 (79, 88) |
| Natural indirect effect: Alcohol use | 1.05 (1.04, 1.06) | 15 (11, 21) | 1.08 (1.07, 1.1) | 16 (12, 21) |
| Total effect | 1.4 (1.29, 1.51) |  | 1.64 (1.47, 1.83) |  |
| *Middle education vs high education* |  |  |  |  |
| Natural direct effect | 1.33 (1.22, 1.45) | 92 (88, 94) | 1.36 (1.21, 1.54) | 90 (85, 93) |
| Natural indirect effect: Alcohol use | 1.03 (1.02, 1.03) | 8 (6, 12) | 1.03 (1.03, 1.04) | 10 (7, 15) |
| Total effect (TE) | 1.37 (1.25, 1.49) |  | 1.41 (1.25, 1.59) |  |
|  |  |  |  |  |
| *Smoking* |  |  |  |  |
| *Low education vs high education* |  |  |  |  |
| Natural direct effect | 1.27 (1.17, 1.38) | 61 (50, 70) | 1.41 (1.24, 1.6) | 76 (65, 83) |
| Natural indirect effect: Smoking | 1.16 (1.14, 1.18) | 39 (31, 50) | 1.11 (1.1, 1.13) | 24 (18, 33) |
| Total effect | 1.47 (1.36, 1.6) |  | 1.57 (1.39, 1.77) |  |
| *Middle education vs high education* |  |  |  |  |
| Natural direct effect | 1.3 (1.18, 1.42) | 72 (61, 78) | 1.3 (1.13, 1.48) | 77 (60, 85) |
| Natural indirect effect: Smoking | 1.11 (1.09, 1.12) | 28 (22, 38) | 1.08 (1.07, 1.09) | 23 (16, 38) |
| Total effect (TE) | 1.44 (1.31, 1.57) |  | 1.4 (1.22, 1.6) |  |
|  |  |  |  |  |
| *BMI* |  |  |  |  |
| *Low education vs high education* |  |  |  |  |
| Natural direct effect | 1.3 (1.2, 1.41) | 91 (87, 94) | 1.52 (1.36, 1.69) | 96 (94, 98) |
| Natural indirect effect: BMI | 1.03 (1.02, 1.03) | 9 (6, 13) | 1.02 (1.01, 1.03) | 4 (2, 6) |
| Total effect | 1.33 (1.23, 1.44) |  | 1.54 (1.38, 1.72) |  |
| *Middle education vs high education* |  |  |  |  |
| Natural direct effect | 1.32 (1.2, 1.44) | 91 (87, 94) | 1.37 (1.21, 1.54) | 95 (91, 98) |
| Natural indirect effect: BMI | 1.03 (1.02, 1.03) | 9 (6, 13) | 1.02 (1.01, 1.02) | 5 (2, 9) |
| Total effect (TE) | 1.35 (1.24, 1.48) |  | 1.39 (1.24, 1.57) |  |
|  |  |  |  |  |
| *Physical inactivity* |  |  |  |  |
| *Low education vs high education* |  |  |  |  |
| Natural direct effect | 1.29 (1.19, 1.4) | 70 (61, 77) | 1.49 (1.34, 1.67) | 80 (73, 84) |
| Natural indirect effect: physical inactivity | 1.11 (1.1, 1.13) | 30 (23, 40) | 1.11 (1.09, 1.12) | 20 (16, 27) |
| Total effect | 1.43 (1.32, 1.55) |  | 1.65 (1.48, 1.84) |  |
| *Middle education vs high education* |  |  |  |  |
| Natural direct effect | 1.31 (1.2, 1.44) | 84 (77, 88) | 1.35 (1.2, 1.52) | 85 (77, 89) |
| Natural indirect effect: physical inactivity | 1.05 (1.05, 1.06) | 16 (12, 23) | 1.05 (1.05, 1.06) | 15 (11, 23) |
| Total effect (TE) | 1.39 (1.27, 1.52) |  | 1.42 (1.26, 1.6) |  |
