# Supplementary material for: Behavioral risk factors and socioeconomic inequalities in ischemic heart disease mortality in the United States: A causal mediation analysis using record linkage data
Source: PLoS Med. 2024 Sep 17;21(9):e1004455. doi: 10.1371/journal.pmed.1004455 (PMC11407680; doi:10.1371/journal.pmed.1004455)
Supplement: S8 Table — (DOCX) [file pmed.1004455.s013.docx]

**S8 Table.** Sensitivity Analysis 2: Natural Direct and Indirect Effects (Hazard Ratio Scale) of Education on Ischemic Heart Disease Mortality Operating via the Pathways of Alcohol Use, Smoking, BMI, and Physical Inactivity, with Decomposition of Indirect Effects into Differential Exposure and Differential Vulnerability on the Pathway of Alcohol Use.

|  | **Male** |  | **Female** |  |
| --- | --- | --- | --- | --- |
|  | HR (95% CI) | % TE (95% CI) | HR (95% CI) | % TE (95% CI) |
| *Low education vs high education* |  |  |  |  |
| Natural direct effect (NDE) | 1.22 (1.06, 1.39) | 32 (13, 46) | 1.25 (0.96, 1.63) | 33 (-7, 57) |
| Natural indirect effect (NIE) | 1.52 (1.43, 1.61) | 68 (53, 88) | 1.58 (1.43, 1.74) | 67 (47, 99) |
| Alcohol use: differential exposure | 1.1 (1.04, 1.16) | 16 (7, 25) | 1.23 (1.09, 1.38) | 30 (10, 61) |
| Alcohol use: differential vulnerability | 0.97 (0.91, 1.03) | -5 (-14, 5) | 0.91 (0.8, 1.03) | -14 (-40, 3) |
| Smoking: mediated | 1.18 (1.14, 1.22) | 27 (20, 36) | 1.12 (1.06, 1.19) | 17 (7, 32) |
| BMI: mediated | 1.03 (1.02, 1.05) | 5 (3, 8) | 1.04 (1.02, 1.07) | 6 (2, 11) |
| Physical inactivity: mediated | 1.16 (1.13, 1.2) | 24 (19, 32) | 1.21 (1.17, 1.26) | 28 (19, 43) |
| Total effect (TE) | 1.85 (1.63, 2.09) |  | 1.98 (1.62, 2.42) |  |
| *Middle education vs high education* |  |  |  |  |
| Natural direct effect (NDE) | 1.29 (1.11, 1.5) | 51 (29, 63) | 1.21 (0.91, 1.62) | 43 (-48, 71) |
| Natural indirect effect (NIE) | 1.28 (1.24, 1.33) | 49 (37, 71) | 1.28 (1.21, 1.36) | 57 (33, 133) |
| Alcohol use: differential exposure | 1.05 (1.02, 1.08) | 10 (4, 18) | 1.09 (1.04, 1.14) | 19 (6, 59) |
| Alcohol use: differential vulnerability | 0.99 (0.95, 1.02) | -2 (-10, 5) | 0.95 (0.91, 1) | -11 (-42, 0) |
| Smoking: mediated | 1.12 (1.09, 1.14) | 21 (15, 32) | 1.09 (1.05, 1.12) | 19 (8, 54) |
| BMI: mediated | 1.03 (1.02, 1.04) | 6 (3, 9) | 1.03 (1.01, 1.05) | 8 (2, 23) |
| Physical inactivity: mediated | 1.08 (1.06, 1.09) | 15 (11, 22) | 1.1 (1.08, 1.13) | 23 (13, 56) |
| Total effect (TE) | 1.66 (1.43, 1.92) |  | 1.56 (1.21, 2) |  |
