# Supplementary material for: Behavioral risk factors and socioeconomic inequalities in ischemic heart disease mortality in the United States: A causal mediation analysis using record linkage data
Source: PLoS Med. 2024 Sep 17;21(9):e1004455. doi: 10.1371/journal.pmed.1004455 (PMC11407680; doi:10.1371/journal.pmed.1004455)
Supplement: S9 Table — (DOCX) [file pmed.1004455.s014.docx]

**S9 Table.** Sensitivity Analysis 3: Natural Direct and Indirect Effects (Hazard Ratio Scale) of Family Income on Ischemic Heart Disease Mortality Operating via the Pathways of Alcohol Use, Smoking, BMI, and Physical Inactivity.

|  | **Male** |  | **Female** |  |
| --- | --- | --- | --- | --- |
|  | HR (95% CI) | % TE (95% CI) | HR (95% CI) | % TE (95% CI) |
| *Low income vs high income* |  |  |  |  |
| Natural direct effect (NDE) | 1.57 (1.43, 1.72) | 58 (51, 64) | 1.54 (1.35, 1.75) | 54 (44, 62) |
| Natural indirect effect (NIE) | 1.38 (1.34, 1.42) | 42 (36, 49) | 1.45 (1.39, 1.5) | 46 (39, 56) |
| Alcohol use | 1.07 (1.06, 1.09) | 9 (7, 11) | 1.12 (1.1, 1.14) | 14 (11, 18) |
| Smoking | 1.11 (1.1, 1.13) | 14 (12, 17) | 1.07 (1.06, 1.09) | 9 (7, 11) |
| BMI | 1.01 (1.01, 1.02) | 2 (1, 2) | 1.03 (1.02, 1.04) | 4 (2, 5) |
| Physical inactivity | 1.14 (1.12, 1.16) | 17 (15, 21) | 1.17 (1.15, 1.19) | 20 (16, 24) |
| Total effect (TE) | 2.17 (1.99, 2.37) |  | 2.22 (1.97, 2.51) |  |
| *Middle income vs high income* |  |  |  |  |
| Natural direct effect (NDE) | 1.31 (1.2, 1.43) | 60 (50, 68) | 1.28 (1.12, 1.46) | 56 (35, 67) |
| Natural indirect effect (NIE) | 1.2 (1.18, 1.21) | 40 (33, 50) | 1.21 (1.19, 1.23) | 44 (33, 63) |
| Alcohol use | 1.04 (1.03, 1.05) | 9 (6, 11) | 1.06 (1.05, 1.07) | 14 (10, 21) |
| Smoking | 1.06 (1.05, 1.06) | 12 (10, 16) | 1.03 (1.03, 1.04) | 7 (5, 11) |
| BMI | 1.01 (1.01, 1.01) | 3 (2, 3) | 1.02 (1.01, 1.02) | 4 (3, 7) |
| Physical inactivity | 1.08 (1.07, 1.09) | 17 (13, 21) | 1.09 (1.08, 1.1) | 19 (14, 28) |
| Total effect (TE) | 1.56 (1.43, 1.7) |  | 1.55 (1.36, 1.77) |  |
