# Supplementary material for: Behavioral risk factors and socioeconomic inequalities in ischemic heart disease mortality in the United States: A causal mediation analysis using record linkage data
Source: PLoS Med. 2024 Sep 17;21(9):e1004455. doi: 10.1371/journal.pmed.1004455 (PMC11407680; doi:10.1371/journal.pmed.1004455)
Supplement: S10 Table — (DOCX) [file pmed.1004455.s015.docx]

**S10 Table.** Descriptive Statistics of Study Participants Aged 25 Years and Older, Stratified by Sex and Decades-Based Birth Cohort-Specific Education Tertiles (Unweighted Sample Sizes; and Weighted Mean, SD, and %).

|  |  |  | **Male (N=233,543, 48.5%)** | | |  | **Female (N=290,492, 51.5%)** | | |
| --- | --- | --- | --- | --- | --- | --- | --- | --- | --- |
|  |  | **Overall** | **Low education** | **Middle education** | **High education** |  | **Low education** | **Middle education** | **High education** |
| Sample size, n (%)^1^ |  | 524,035 (100) | 97,825 (40.8) | 75,935 (32.6) | 59,783 (26.6) |  | 119,116 (39.1) | 103,934 (36.4) | 67,442 (24.5) |
| Age at survey, mean (SD) |  | 50.3 (16.2) | 48.2 (15.3) | 47.8 (16.3) | 53.8 (14.7) |  | 51.0 (16.3) | 49.6 (17.5) | 52.8 (15.3) |
| Years follow up, mean (SD) |  | 10.3 (6.1) | 10.3 (6.1) | 9.9 (6.0) | 10.5 (6.2) |  | 10.6 (6.2) | 9.9 (5.9) | 10.4 (6.2) |
| Person-years |  | 5,471,915 | 1,019,166 | 752,066 | 631,126 |  | 1,305,763 | 1,047,877 | 715,917 |
| Alcohol use, mean grams per daily (SD) in all participants |  | 5.7 (19.0) | 9.2 (28.8) | 8.3 (20.7) | 7.6 (21.3) |  | 2.5 (12.3) | 3.3 (9.9) | 3.9 (10.1) |
| Sample size of current drinkers, n (%)^2^ |  | 325,601 (64.7) | 61,932 (64.6) | 55,158 (73.6) | 45,291 (77.0) |  | 52949 (47.3) | 63825 (63.4) | 46446 (70.7) |
| Alcohol use, mean grams per day (SD) in current drinkers only |  | 8.95 (23.0) | 14.3 (34.8) | 11.2 (23.4) | 9.8 (23.8) |  | 5.3 (17.4) | 5.2 (12.0) | 5.5 (11.7) |
| Alcohol use, n (%)^2^ |  |  |  |  |  |  |  |  |  |
| Lifetime abstainer |  | 160,725 (28.6) | 24,844 (25.0) | 14,407 (18.7) | 10,666 (17.2) |  | 58,636 (46.7) | 34,330 (31.4) | 17,842 (25.1) |
| Former drinker |  | 37,709 (6.8) | 11,049 (10.4) | 6,370 (7.7) | 3,826 (5.8) |  | 7,531 (6.0) | 5,779 (5.2) | 3,154 (4.2) |
| Category I: (0, 20] g/day |  | 287,928 (57.3) | 49,317 (52.0) | 45,809 (61.9) | 38,925 (66.6) |  | 49,693 (44.4) | 60,291 (59.9) | 43,893 (66.8) |
| Category II: (20, 40] g/day for male; >20 g/day for female |  | 26,742 (5.3) | 6,810 (6.9) | 6,004 (7.6) | 4,585 (7.6) |  | 3,256 (2.8) | 3,534 (3.5) | 2,553 (3.9) |
| Category III: (40, 60] g/day for male only |  | 5,687 (1.1) | 2,715 (2.7) | 1,805 (2.3) | 1,167 (1.9) |  | 0 (0.0) | 0 (0.0) | 0 (0.0) |
| Category IV: >60 g/day for male only |  | 5,244 (1.0) | 3,090 (3.1) | 1,540 (1.8) | 614 (0.9) |  | 0 (0.0) | 0 (0.0) | 0 (0.0) |
| Smoking, n (%)^2^ |  |  |  |  |  |  |  |  |  |
| Never smoker |  | 292,254 (56.0) | 39,389 (40.9) | 37,116 (50.4) | 36,146 (62.2) |  | 69,442 (57.2) | 63,419 (61.6) | 46,742 (70.6) |
| Former smoker |  | 127,703 (24.6) | 27,964 (28.0) | 22,680 (29.6) | 17,779 (29.1) |  | 21,490 (18.6) | 22,631 (21.7) | 15,159 (21.8) |
| Current someday smoker |  | 21,401 (3.8) | 5,632 (5.5) | 3,813 (4.7) | 1,662 (2.5) |  | 4,630 (3.6) | 4,022 (3.6) | 1,642 (2.3) |
| Current everyday smoker |  | 82,677 (15.6) | 24,840 (25.7) | 12,326 (15.4) | 4,196 (6.2) |  | 23,554 (20.6) | 13,862 (13.1) | 3,899 (5.3) |
| BMI, n (%)^2^ |  |  |  |  |  |  |  |  |  |
| Underweight |  | 8,724 (1.5) | 26,409 (25.9) | 20,489 (25.6) | 18,993 (30.5) |  | 39,814 (34.1) | 41,789 (41.1) | 33,567 (50.9) |
| Healthy weight |  | 181,061 (34.1) | 901 (0.9) | 473 (0.6) | 298 (0.4) |  | 2,634 (2.2) | 2,505 (2.4) | 1,913 (2.7) |
| Overweight |  | 187,626 (36.3) | 41,148 (41.7) | 33,270 (44) | 27,713 (47.0) |  | 37,224 (30.7) | 30,021 (28.7) | 18,250 (27.1) |
| Obese |  | 146,624 (28.1) | 29,367 (31.4) | 21,703 (29.7) | 12,779 (22.1) |  | 39,444 (33.0) | 29,619 (27.9) | 13,712 (19.4) |
| Physical inactivity, n (%)^2^ |  |  |  |  |  |  |  |  |  |
| Active |  | 226,244 (45.0) | 14,346 (15.1) | 12,504 (16.9) | 10,287 (17.6) |  | 21,593 (18.9) | 21,890 (21.4) | 14,248 (21.6) |
| Somewhat active |  | 94,868 (18.5) | 47,956 (47.5) | 23,743 (30.3) | 13,064 (20.8) |  | 63,429 (50.7) | 37,593 (34.2) | 17,138 (23.8) |
| Sedentary |  | 202,923 (36.5) | 35,523 (37.4) | 39,688 (52.8) | 36,432 (61.6) |  | 34,094 (30.4) | 44,451 (44.4) | 36,056 (54.6) |
| Race/ethnicity, n (%)^2^ |  |  |  |  |  |  |  |  |  |
| White |  | 342,084 (70.7) | 55,671 (62.4) | 54,214 (74.6) | 46,510 (80.3) |  | 63,021 (61.3) | 71,626 (74.0) | 51,042 (78.7) |
| Black |  | 72,601 (11.3) | 14,808 (12.9) | 9,406 (10.8) | 4,345 (6.0) |  | 21,537 (14.6) | 15,700 (12.1) | 6,805 (8.0) |
| Hispanic |  | 81,415 (12.6) | 23,835 (21.1) | 8,153 (9.5) | 3,711 (5.2) |  | 29,746 (19.6) | 11,593 (8.9) | 4,377 (5.1) |
| Other |  | 27,935 (5.5) | 3,511 (3.6) | 4,162 (5.1) | 5,217 (8.4) |  | 4,812 (4.5) | 5,015 (4.9) | 5,218 (8.2) |
| Income, n (%)^2^ |  |  |  |  |  |  |  |  |  |
| Low |  | 142,955 (22.4) | 17,515 (20.7) | 27,756 (39.9) | 34,064 (60.0) |  | 14,717 (16.5) | 28,584 (32.8) | 34,451 (55.9) |
| Middle |  | 128,998 (25.0) | 35,693 (32.5) | 13,510 (15.0) | 5,093 (6.8) |  | 54,604 (38.6) | 26,952 (20.9) | 7,103 (8.1) |
| High |  | 157,087 (34.7) | 27,050 (28.7) | 22,166 (28.7) | 10,470 (16.5) |  | 25,957 (24.8) | 29,703 (28.6) | 13,652 (18.3) |
| Missing |  | 94,995 (17.9) | 17,567 (18.2) | 12,503 (16.3) | 10,156 (16.7) |  | 23,838 (20.1) | 18,695 (17.7) | 12,236 (17.7) |
| Marital status, n (%)^2^ |  |  |  |  |  |  |  |  |  |
| Married/cohabitating |  | 286,008 (67.9) | 57,339 (69.2) | 44,103 (70.5) | 39,760 (79.1) |  | 57,431 (61.3) | 50,918 (62.9) | 36,457 (68.8) |
| Not married/cohabitating |  | 238,027 (32.1) | 40,486 (30.8) | 31,832 (29.5) | 20,023 (20.9) |  | 61,685 (38.7) | 53,016 (37.1) | 30,985 (31.2) |

^1^ Percentages of the overall sample and educational levels among male and female participants, respectively.

^2^ Percentages by column.
