# Supplementary material for: Behavioral risk factors and socioeconomic inequalities in ischemic heart disease mortality in the United States: A causal mediation analysis using record linkage data
Source: PLoS Med. 2024 Sep 17;21(9):e1004455. doi: 10.1371/journal.pmed.1004455 (PMC11407680; doi:10.1371/journal.pmed.1004455)
Supplement: S11 Table — (DOCX) [file pmed.1004455.s016.docx]

**S11 Table.** Sample Size (Unweighted n) and Proportion (Weighted %) by Decades-Based Birth Cohort and Educational Level.

|  | Overall | <=1920 | 1921-1930 | 1931-1940 | 1941-1950 | 1951-1960 | 1961-1970 | 1971-1980 | 1981-1990 | >=1991 |
| --- | --- | --- | --- | --- | --- | --- | --- | --- | --- | --- |
| Sample size, n | 524,035 | 14,876 | 36,585 | 54,040 | 85,952 | 107,452 | 107,424 | 80,119 | 36,568 | 1,019 |
| Educational level for main analysis, n (%) | | | | | | | | | | |
| Low (high school or less) | 231,685 (41.9) | 10,336 (68.2) | 23,514 (62.8) | 30,748 (55.0) | 37,418 (42.3) | 44,811 (40.1) | 43,472 (38.8) | 29,672 (35.6) | 11,426 (31.5) | 288 (32.8) |
| Middle (some college) | 146,483 (28.0) | 2,647 (18.0) | 7,282 (20.2) | 12,341 (22.9) | 24,094 (27.6) | 32,468 (29.8) | 31,846 (29.2) | 24,019 (29.6) | 11,461 (31.3) | 325 (31.2) |
| High (Bachelor’s degree or more) | 145,867 (30.1) | 1,893 (13.8) | 5,789 (17.1) | 10,951 (22.1) | 24,440 (30.2) | 30,173 (30.1) | 32,106 (31.9) | 26,428 (34.8) | 13,681 (37.3) | 406 (35.9) |
|  | | | | | | | | | | |
| Educational level based on birth cohort-specific education tertiles^1^ for sensitivity analysis, n (%) | | | | | | | | | | |
| Low | 216,941 (39.9) | 5,551 (35.1) | 17,047 (44.1) | 19,994 (34.3) | 37,418 (42.3) | 44,811 (40.1) | 43,472 (38.8) | 29,672 (35.6) | 18,497 (50.6) | 479 (50.3) |
| Middle | 179,869 (34.6) | 4,785 (33.1) | 11,567 (32.7) | 19,164 (36.1) | 24,094 (27.6) | 32,468 (29.8) | 31,846 (29.2) | 41,506 (52.3) | 13,980 (38.4) | 459 (43.6) |
| High | 127,225 (25.6) | 4,540 (31.8) | 7,971 (23.2) | 14,882 (29.6) | 24,440 (30.2) | 30,173 (30.1) | 32,106 (31.9) | 8,941 (11.8) | 4,091 (11.1) | 81 (6.1) |

^1^ Survey weights were accounted for when estimating tertiles for each birth cohort.
