# Supplementary material for: Behavioral risk factors and socioeconomic inequalities in ischemic heart disease mortality in the United States: A causal mediation analysis using record linkage data
Source: PLoS Med. 2024 Sep 17;21(9):e1004455. doi: 10.1371/journal.pmed.1004455 (PMC11407680; doi:10.1371/journal.pmed.1004455)
Supplement: S12 Table — (DOCX) [file pmed.1004455.s017.docx]

**S12 Table.** Tests of Proportional Hazards Assumptions for Cox Proportional Hazards Models Using Education Defined by Decades-Based Birth Cohort-Specific Education Tertiles.

|  | Male | | | | | | Female | | | | | |
| --- | --- | --- | --- | --- | --- | --- | --- | --- | --- | --- | --- | --- |
|  | Minimally adjusted | | | Fully adjusted | | | Minimally adjusted | | | Fully adjusted | | |
|  | Chi-square statistics | Degrees of freedom | p-value | Chi-square statistics | Degrees of freedom | p-value | Chi-square statistics | Degrees of freedom | p-value | Chi-square statistics | Degrees of freedom | p-value |
| *Educational level based on birth cohort-specific education tertiles for sensitivity analysis* | | | | | | | | | | | | |
| Education | 0.000405 | 2 | 1 | 0.000235 | 2 | 1 | 0.000284 | 2 | 1 | 0.000159 | 2 | 1 |
| Marital status | 0.00024 | 1 | 0.99 | 0.000213 | 1 | 0.99 | 0.000165 | 1 | 0.99 | 0.000129 | 1 | 0.99 |
| Race and ethnicity | 0.000169 | 3 | 1 | 0.000215 | 3 | 1 | 0.000172 | 3 | 1 | 0.000168 | 3 | 1 |
| Alcohol use | 0.000145 | 21 | 1 | 0.000193 | 5 | 1 | 5.39E-05 | 21 | 1 | 0.000205 | 3 | 1 |
| Smoking | 0.000846 | 27 | 1 | 0.000248 | 3 | 1 | 0.000593 | 27 | 1 | 0.000152 | 3 | 1 |
| BMI |  |  |  | 9.47E-05 | 3 | 1 |  |  |  | 0.000168 | 3 | 1 |
| Physical inactivity |  |  |  | 8.64E-05 | 2 | 1 |  |  |  | 0.000254 | 2 | 1 |
| Survey year |  |  |  | 0.000188 | 21 | 1 |  |  |  | 5.23E-05 | 21 | 1 |
| Global test |  |  |  | 0.001136 | 40 | 1 |  |  |  | 0.000988 | 38 | 1 |
