# Supplementary material for: Behavioral risk factors and socioeconomic inequalities in ischemic heart disease mortality in the United States: A causal mediation analysis using record linkage data
Source: PLoS Med. 2024 Sep 17;21(9):e1004455. doi: 10.1371/journal.pmed.1004455 (PMC11407680; doi:10.1371/journal.pmed.1004455)
Supplement: S13 Table — (DOCX) [file pmed.1004455.s018.docx]

**S13 Table.** Associations Between Education Defined by Decades-Based Birth Cohort-Specific Education Tertiles, Alcohol Use, Smoking, BMI, Physical inactivity, and Ischemic Heart Disease Mortality in Sex-stratified Cox Proportional Hazards Models.

|  | Male | | | | | | Female | | | | | |
| --- | --- | --- | --- | --- | --- | --- | --- | --- | --- | --- | --- | --- |
|  | Minimally adjusted | | | Fully adjusted | | | Minimally adjusted | | | Fully adjusted | | |
|  | HR | 95% CI | p-value | HR | 95% CI | p-value | HR | 95% CI | p-value | HR | 95% CI | p-value |
| Education |  |  |  |  |  |  |  |  |  |  |  |  |
| Low | 1.72 | (1.61, 1.85) | <.001 | 1.23 | (1.14, 1.32) | <.001 | 1.75 | (1.61, 1.89) | <.001 | 1.34 | (1.24, 1.46) | <.001 |
| Middle | 1.43 | (1.33, 1.54) | <.001 | 1.17 | (1.09, 1.26) | <.001 | 1.39 | (1.27, 1.51) | <.001 | 1.2 | (1.1, 1.31) | <.001 |
| High | ref |  |  | ref |  |  | ref |  |  | ref |  |  |
| Marital status |  |  |  |  |  |  |  |  |  |  |  |  |
| Not married/cohabitating | ref |  |  | ref |  |  | ref |  |  | ref |  |  |
| Married/cohabitating | 0.63 | (0.6, 0.67) | <.001 | 0.67 | (0.64, 0.71) | <.001 | 0.63 | (0.59, 0.68) | <.001 | 0.7 | (0.65, 0.76) | <.001 |
| Race and ethnicity |  |  |  |  |  |  |  |  |  |  |  |  |
| White | ref |  |  | ref |  |  | ref |  |  | ref |  |  |
| Black | 0.93 | (0.85, 1.02) | 0.167 | 0.87 | (0.79, 0.96) | 0.005 | 1.13 | (1.02, 1.25) | 0.016 | 1.01 | (0.91, 1.11) | 0.89 |
| Hispanic | 0.73 | (0.66, 0.81) | <.001 | 0.76 | (0.68, 0.84) | <.001 | 0.77 | (0.69, 0.87) | <.001 | 0.76 | (0.68, 0.86) | <.001 |
| Other | 0.73 | (0.6, 0.89) | 0.002 | 0.73 | (0.6, 0.88) | 0.001 | 0.69 | (0.57, 0.83) | <.001 | 0.73 | (0.6, 0.88) | 0.001 |
| Alcohol use |  |  |  |  |  |  |  |  |  |  |  |  |
| Lifetime abstainer |  |  |  | ref |  |  |  |  |  | ref |  |  |
| Former drinker |  |  |  | 1.04 | (0.96, 1.14) | 0.368 |  |  |  | 1.07 | (0.96, 1.19) | 0.223 |
| Category I: (0, 20] g/day |  |  |  | 0.74 | (0.69, 0.8) | <.001 |  |  |  | 0.65 | (0.61, 0.7) | <.001 |
| Category II: (20, 40] g/day for male; >20 g/day for female |  |  |  | 0.73 | (0.65, 0.83) | <.001 |  |  |  | 0.61 | (0.49, 0.76) | <.001 |
| Category III: (40, 60] g/day for male only |  |  |  | 0.91 | (0.75, 1.09) | 0.289 |  |  |  | - |  |  |
| Category IV: >60 g/day for male only |  |  |  | 1.08 | (0.87, 1.33) | 0.487 |  |  |  | - |  |  |
| Smoking |  |  |  |  |  |  |  |  |  |  |  |  |
| Never smoker |  |  |  | ref |  |  |  |  |  | ref |  |  |
| Former smoker |  |  |  | 1.42 | (1.32, 1.53) | <.001 |  |  |  | 1.43 | (1.33, 1.53) | <.001 |
| Current someday smoker |  |  |  | 1.77 | (1.5, 2.08) | <.001 |  |  |  | 1.99 | (1.62, 2.44) | <.001 |
| Current everyday smoker |  |  |  | 2.46 | (2.25, 2.68) | <.001 |  |  |  | 2.34 | (2.12, 2.58) | <.001 |
| BMI |  |  |  |  |  |  |  |  |  |  |  |  |
| Underweight |  |  |  | 1.51 | (1.16, 1.96) | 0.002 |  |  |  | 1.29 | (1.06, 1.56) | 0.01 |
| Healthy weight |  |  |  | ref |  |  |  |  |  | ref |  |  |
| Overweight |  |  |  | 0.97 | (0.9, 1.05) | 0.452 |  |  |  | 1.02 | (0.95, 1.1) | 0.548 |
| Obese |  |  |  | 1.37 | (1.27, 1.48) | <.001 |  |  |  | 1.29 | (1.2, 1.39) | <.001 |
| Physical inactivity |  |  |  |  |  |  |  |  |  |  |  |  |
| Active |  |  |  | ref |  |  |  |  |  | ref |  |  |
| Somewhat active |  |  |  | 1.28 | (1.51, 1.73) | <.001 |  |  |  | 1.33 | (1.19, 1.48) | <.001 |
| Sedentary |  |  |  | 1.62 | (1.18, 1.4) | <.001 |  |  |  | 1.85 | (1.69, 2.02) | <.001 |
