# Supplementary material for: Behavioral risk factors and socioeconomic inequalities in ischemic heart disease mortality in the United States: A causal mediation analysis using record linkage data
Source: PLoS Med. 2024 Sep 17;21(9):e1004455. doi: 10.1371/journal.pmed.1004455 (PMC11407680; doi:10.1371/journal.pmed.1004455)
Supplement: S14 Table — (DOCX) [file pmed.1004455.s019.docx]

**S14 Table.** Sensitivity Analysis 4: Natural Direct and Indirect Effects (Hazard Ratio Scale) of Educational Level Based on Birth Cohort-Specific Education Tertiles.

|  | **Male** |  | **Female** |  |
| --- | --- | --- | --- | --- |
|  | HR (95% CI) | % TE (95% CI) | HR (95% CI) | % TE (95% CI) |
| *Low education vs high education* |  |  |  |  |
| Natural direct effect (NDE) | 1.11 (1.03, 1.21) | 21 (5, 33) | 1.19 (1.08, 1.31) | 32 (16, 44) |
| Natural indirect effect (NIE) | 1.52 (1.46, 1.57) | 79 (67, 95) | 1.45 (1.39, 1.5) | 68 (57, 83) |
| Alcohol use | 1.07 (1.06, 1.09) | 13 (10, 17) | 1.09 (1.08, 1.11) | 16 (13, 21) |
| Smoking | 1.18 (1.16, 1.2) | 31 (26, 38) | 1.11 (1.09, 1.13) | 19 (14, 24) |
| BMI | 1.03 (1.02, 1.04) | 5 (4, 7) | 1.03 (1.02, 1.04) | 5 (3, 7) |
| Physical inactivity | 1.16 (1.14, 1.19) | 29 (24, 35) | 1.17 (1.15, 1.19) | 28 (23, 35) |
| Total effect (TE) | 1.69 (1.57, 1.82) | 100 | 1.72 (1.57, 1.88) | 100 |
| *Middle education vs. high education* |  |  |  |  |
| Natural direct effect (NDE) | 1.13 (1.04, 1.23) | 37 (14, 51) | 1.13 (1.02, 1.26) | 40 (8, 56) |
| Natural indirect effect (NIE) | 1.24 (1.21, 1.26) | 63 (49, 85) | 1.21 (1.18, 1.23) | 60 (45, 91) |
| Alcohol use | 1.03 (1.03, 1.04) | 10 (7, 14) | 1.04 (1.03, 1.05) | 13 (9, 21) |
| Smoking | 1.09 (1.08, 1.1) | 26 (20, 36) | 1.05 (1.04, 1.06) | 16 (11, 26) |
| BMI | 1.02 (1.01, 1.02) | 5 (4, 8) | 1.02 (1.01, 1.02) | 5 (3, 8) |
| Physical inactivity | 1.08 (1.07, 1.09) | 22 (17, 30) | 1.08 (1.07, 1.09) | 26 (19, 39) |
| Total effect (TE) | 1.39 (1.28, 1.52) | 100 | 1.36 (1.23, 1.51) | 100 |
